# Supplementary material for: Impact of phylogeny on the inference of functional sectors from protein sequence data
Source: PLoS Comput Biol. 2024 Sep 23;20(9):e1012091. doi: 10.1371/journal.pcbi.1012091 (PMC11449291; doi:10.1371/journal.pcbi.1012091)
Supplement: S1 Appendix — (PDF) [file pcbi.1012091.s001.pdf]

# S1 Appendix for “Impact of phylogeny on the inference of functional sectors from protein sequence data”

Nicola Dietler[1,2], Alia Abbara[1,2], Subham Choudhury[1,2], Anne-Florence Bitbol[1,2,*]

**1** Institute of Bioengineering, School of Life Sciences, École Polytechnique Fédérale de Lausanne (EPFL), CH-1015 Lausanne, Switzerland
**2** SIB Swiss Institute of Bioinformatics, CH-1015 Lausanne, Switzerland
*anne-florence.bitbol@epfl.ch

## Contents

**1 Analytical approximation of the inverse covariance matrix at higher orders 1**
1.1 Complex saddle point approximation . . . 1
1.2 High-temperature Plefka expansion of the TAP free energy . . . 2
1.2.1 TAP free energy expansion . . . 3
1.2.2 Extremized magnetizations . . . 3
1.2.3 Inverse covariance matrix . . . 4
1.3 Comparison to synthetic data . . . 4

**2 Impact of selection parameters on mutational effect recovery 6**
2.1 Impact of the favored trait value . . . 6
2.2 Impact of selection strength . . . 10

## 1 Analytical approximation of the inverse covariance matrix at higher orders

### 1.1 Complex saddle point approximation

Consider a sequence of $L$ Ising spins $\{\sigma_l\}$, with $\sigma_l \in \{1, -1\}$ for all $l \in [1, L]$, with Hamiltonian

$$H = \frac{\kappa}{2}\Big(\sum_l \sigma_l D_l - \tau^*\Big)^2 + \sum_l \sigma_l f_l, \tag{S1}$$

where $f_l$ is a site-specific auxiliary field, which we use for calculations and then set to zero. The partition function is given by

$$Z = \sum_{\{\sigma_l\}} \exp(-H) = \sum_{\{\sigma_l\}} \exp\Big(-\frac{\kappa}{2}\Big[\sum_l \sigma_l D_l - \tau^*\Big]^2 - \sum_l \sigma_l f_l\Big). \tag{S2}$$

Then, the magnetization $m_l \equiv \langle \sigma_l \rangle$ at site $l$ reads

$$m_l = -\left.\frac{\partial \ln Z}{\partial f_l}\right|_{\vec{f}=\vec{0}}, \tag{S3}$$

and the covariance $C_{ll'} \equiv \langle \sigma_l \sigma_{l'} \rangle - \langle \sigma_l \rangle \langle \sigma_{l'} \rangle$ reads

$$C_{ll'} = \left.\frac{\partial^2 \ln Z}{\partial f_{l'} \partial f_l}\right|_{\vec{f}=\vec{0}} = -\left.\frac{\partial m_l}{\partial f_{l'}}\right|_{\vec{f}=\vec{0}}. \tag{S4}$$

To compute the partition function $Z$, we employ the Hubbard-Stratonovich transform [1], which involves an auxiliary field $h$:

$$\exp\Big(-\frac{\kappa}{2}x^2\Big) = \frac{1}{\sqrt{2\pi\kappa}} \int_{-\infty}^{\infty} \exp\Big(-\frac{1}{2\kappa}h^2 - ihx\Big)\, dh. \tag{S5}$$

Employing this result, we have

$$
\begin{aligned}
Z &= \frac{1}{\sqrt{2\pi\kappa}} \int_{-\infty}^{\infty} dh \sum_{\{\sigma_l\}} \exp\Big( -\frac{1}{2\kappa}h^2 - ih\Big[\sum_l \sigma_l D_l - \tau^*\Big] - \sum_l \sigma_l f_l \Big) \\
&= \frac{1}{\sqrt{2\pi\kappa}} \int_{-\infty}^{\infty} dh \exp\Big( -\frac{1}{2\kappa}h^2 + ih\tau^* \Big) \prod_l \sum_{\sigma_l} \exp\Big( -ih\sigma_l D_l - \sigma_l f_l \Big) \\
&= \frac{2^L}{\sqrt{2\pi\kappa}} \int_{-\infty}^{\infty} dh \exp\Big( -\frac{1}{2\kappa}h^2 + ih\tau^* \Big) \prod_l \cosh\Big( ihD_l + f_l \Big) \\
&= \frac{2^L}{\sqrt{2\pi\kappa}} \int_{-\infty}^{\infty} dh \exp\left( -\frac{h^2}{2\kappa} \right) a(h),
\end{aligned} \tag{S6}
$$

where we introduced

$$
a(h) \equiv \exp\left[ ih\tau^* + \sum_l \ln\cosh\Big( ihD_l + f_l \Big) \right]. \tag{S7}
$$

The complex saddle point approximation [2] yields the following expansion when $\kappa \to 0$:

$$
Z = 2^L a(0) + \kappa\, 2^{L-1} a^{(2)}(0) + \kappa^2\, 2^{L-3} a^{(4)}(0) + O(\kappa^3)\,, \tag{S8}
$$

where $a^{(i)}$ denotes the $i$-th derivative of $a$ with respect to $h$. Therefore,

$$
\ln Z = \ln\left[2^L a(0)\right] + \frac{\kappa}{2}\frac{a^{(2)}(0)}{a(0)} + \frac{\kappa^2}{8}\left\{ \frac{a^{(4)}(0)}{a(0)} - \left[\frac{a^{(2)}(0)}{a(0)}\right]^2 \right\} + O(\kappa^3)\,. \tag{S9}
$$

Computing the successive derivatives of $a$ (see Eq S7) and employing the definitions in Eq S3 and S4, we obtain

$$
m_k = \tau^* \kappa D_k \left[ 1 + \kappa \left( D_k^2 - \sum_l D_l^2 \right) \right] + O(\kappa^3), \tag{S10}
$$

$$
C_{jk} = -\kappa D_j D_k \left[ 1 + \kappa \left( D_k^2 + D_j^2 - \sum_l D_l^2 \right) \right] (1 - \delta_{jk}) + \Big( 1 - {\tau^*}^2 \kappa^2 D_k^2 \Big) \delta_{jk} + O(\kappa^3). \tag{S11}
$$

(S12)

We also obtain the elements of the inverse correlation matrix:

$$
C^{-1}_{jk} = \kappa D_j D_k (1 - \delta_{jk}) + \left[ 1 + \kappa^2 D_j^2 \left( \sum_l D_l^2 - D_j^2 + {\tau^*}^2 \right) \right] \delta_{jk} + O(\kappa^3). \tag{S13}
$$

To first order in $\kappa$, we recover the results of our previous work [3], where we employed the mean-field approximation to first order in the coupling strengths $\kappa D_k D_l$. Moreover, the ICOD matrix reads

$$
\tilde{C}^{-1}_{jk} = \kappa D_j D_k (1 - \delta_{jk}) + O(\kappa^3)\,, \tag{S14}
$$

i.e. our previous expression [3] holds to second order in $\kappa$, generalizing our previous results.

## 1.2 High-temperature Plefka expansion of the TAP free energy

We can derive the expression of the inverse covariance matrix in the large temperature limit (or equivalently here, in the limit of small coupling $\kappa$), using a *Plefka expansion*. We follow the calculation from [4], starting from the Hamiltonian in Eq S1 with no auxiliary field, and ignoring additive constant terms:

$$
H = \frac{\kappa}{2} \sum_{i,j} D_i D_j \sigma_i \sigma_j - \kappa \sum_j \tau^* \sigma_j D_j \tag{S15}
$$

$$
= \frac{\tilde{\kappa}}{2} \sum_{i,j} \tilde{D}_i \tilde{D}_j \sigma_i \sigma_j - \tilde{\kappa} \sum_j \tilde{\tau} \sigma_j \tilde{D}_j, \tag{S16}
$$

where we defined rescaled parameters as

$$\tilde{\kappa} = \kappa \sum_j D_j^2, \tag{S17}$$

$$\tilde{\tau} = \frac{\tau^*}{\sqrt{\sum_j D_j^2}}, \tag{S18}$$

$$\tilde{D}_i = \frac{D_i}{\sqrt{\sum_j D_j^2}}. \tag{S19}$$

For simplicity and to match notations from [4], we introduce $J_{ij} = -\tilde{D}_i \tilde{D}_j$, and $h_i = -\tilde{\tau}\tilde{D}_j$.

### 1.2.1 TAP free energy expansion

We are interested in the perturbative expansion in $\tilde{\kappa}$ of the *TAP free energy*, which is the free energy $\Phi = \log Z$ constrained to fixed values of the magnetizations $m_i = \langle \sigma_i \rangle$ and variances $v_i = \langle (\sigma_i - m_i)^2 \rangle$, by using Lagrange multipliers. Focusing on binary spins, we have $v_i = 1 - m_i^2$. Ref. [4] suggested a full expression of the perturbative expansion, and proved that it is exact up to order 4. This expansion reads:

$$\Phi(\tilde{\kappa}) = \Phi(0) + \frac{\tilde{\kappa}}{2} \sum_{i,j} J_{ij} m_i m_j - \tilde{\kappa} \sum_i h_i m_i + \sum_{p=1}^{4} \frac{\tilde{\kappa}^p}{2p} \sum_{\substack{i_1,\ldots,i_p \\ \text{pairwise distinct}}} J_{i_1 i_2} \ldots J_{i_p i_1} \prod_{\alpha=1}^{p} v_{i_\alpha}, \tag{S20}$$

where

$$\Phi(0) = -\sum_i \left[ \frac{1 - m_i}{2} \log\left(\frac{1 - m_i}{2}\right) + \frac{1 + m_i}{2} \log\left(\frac{1 + m_i}{2}\right) \right]. \tag{S21}$$

### 1.2.2 Extremized magnetizations

The first step is then to extremize this free energy with respect to magnetizations:

$$\begin{aligned} \frac{\partial \Phi}{\partial m_i} = \frac{1}{2} \log \frac{1 + m_i}{1 - m_i} + \tilde{\kappa} \sum_j J_{ij} m_j - \tilde{\kappa} h_i - \tilde{\kappa}^2 \sum_{\substack{j \\ j \neq i}} J_{ij}^2 (1 - m_j^2) m_i \\ - \tilde{\kappa}^3 \sum_{\substack{j \neq k \\ j,k \neq i}} J_{ij} J_{jk} J_{ki} (1 - m_j^2)(1 - m_k^2) m_i - \tilde{\kappa}^4 \sum_{\substack{j \neq k \neq l \\ j,k,l \neq i}} J_{ij} J_{jk} J_{kl} J_{li} (1 - m_j^2)(1 - m_k^2)(1 - m_l^2) m_i = 0. \end{aligned} \tag{S22}$$

We write the perturbative expansion $m_i = m_i^{(0)} + \tilde{\kappa} m_i^{(1)} + \tilde{\kappa}^2 m_i^{(2)} + \tilde{\kappa}^3 m_i^{(3)} + \tilde{\kappa}^4 m_i^{(4)}$, where we only keep leading terms in $L$ (in the large $L$ limit) in all $m_i^{(k)}$. Inserting this expression in S22, we obtain

$$m_i^{(0)} = 0 \tag{S23a}$$

$$m_i^{(1)} = -h_i = \tilde{\tau} \tilde{D}_i \tag{S23b}$$

$$m_i^{(2)} = \sum_j J_{ij} m_j^{(1)} = -\tilde{\tau} \tilde{D}_i \sum_j \tilde{D}_j^2 \tag{S23c}$$

$$m_i^{(3)} = \sum_j J_{ij} m_j^{(2)} = \tilde{\tau} \tilde{D}_i \left( \sum_j \tilde{D}_j^2 \right)^2 \tag{S23d}$$

$$m_i^{(4)} = \sum_j J_{ij} m_j^{(3)} = -\tilde{\tau} \tilde{D}_i \left( \sum_j \tilde{D}_j^2 \right)^3. \tag{S23e}$$

Note that the dominant terms we retained are of order $1/\sqrt{L}$.

### 1.2.3 Inverse covariance matrix

From the free energy, we can derive the elements of the inverse covariance matrix through $C^{-1}_{ij} = -\dfrac{\partial^2 \Phi}{\partial m_i \partial m_j}$. Differentiating Eq S22 yields

$$\begin{aligned}
C^{-1}_{ii} =& \frac{1}{2}\left(\frac{1}{1+m_i} + \frac{1}{1-m_i}\right) + \tilde{\kappa}^2 \sum_{\substack{j \\ j\neq i}} J_{ij}^2 (1-m_j^2) + \tilde{\kappa}^3 \sum_{\substack{j\neq k \\ j,k\neq i}} J_{ij}J_{jk}J_{ki}(1-m_j^2)(1-m_k^2) \\
&+ \tilde{\kappa}^4 \sum_{\substack{j\neq k\neq l \\ j,k,l\neq i}} J_{ij}J_{jk}J_{kl}J_{li}(1-m_j^2)(1-m_k^2)(1-m_l^2) + O(\tilde{\kappa}^5), \qquad \text{(S24)} \\
C^{-1}_{ij} =& -\tilde{\kappa} J_{ij} - 2\tilde{\kappa}^2 J_{ij}^2 m_i m_j - 4\tilde{\kappa}^3 \sum_{\substack{k \\ k\neq j,i}} J_{ij}J_{jk}J_{ki}(1-m_k^2) m_i m_j \\
&- 6\tilde{\kappa}^4 \sum_{\substack{k\neq l \\ k,l\neq i,j}} J_{ij}J_{jk}J_{kl}J_{li}(1-m_k^2)(1-m_l^2) m_i m_j + O(\tilde{\kappa}^5) \;\text{ if } i\neq j. \qquad \text{(S25)}
\end{aligned}$$

Injecting Eq S23 finally yields, up to order 4 in $\tilde{\kappa}$:

$$\begin{aligned}
C^{-1}_{ii} =& 1 + \tilde{\kappa}^2 \tilde{D}_i^2 \left(\tilde{\tau}^2 + \sum_{\substack{j \\ j\neq i}} \tilde{D}_j^2\right) - \tilde{\kappa}^3 \tilde{D}_i^2 \left(2\tilde{\tau}^2 \sum_j \tilde{D}_j^2 + \sum_{\substack{j\neq k \\ j,k\neq i}} \tilde{D}_j^2 \tilde{D}_k^2\right) \\
&+ \tilde{\kappa}^4 \tilde{D}_i^2 \left(\tilde{\tau}^4 \tilde{D}_i^2 + 3\tilde{\tau}^2 (\sum_k \tilde{D}_k^2)^2 + \sum_{\substack{j\neq k\neq l \\ j,k,l\neq i}} \tilde{D}_j^2 \tilde{D}_k^2 \tilde{D}_l^2\right) + O(\tilde{\kappa}^5)\,, \qquad \text{(S26)} \\
C^{-1}_{ij} =& \tilde{\kappa} \tilde{D}_i \tilde{D}_j + O(\tilde{\kappa}^5) \;\text{ if } i\neq j. \qquad \text{(S27)}
\end{aligned}$$

Except the 1 in the first line, all other terms are of order $1/L$. These expressions are consistent with the second order expansion in $\kappa$ of the inverse correlation matrix obtained in Eq S13. They further extend our previous results on the ICOD matrix [3], up to order 4 in $\tilde{\kappa}$.

## 1.3 Comparison to synthetic data

In Fig I, we compare our analytical approximations from Eq S26, S27 to the elements of the inverse covariance matrix obtained directly from synthetic data. We find a good agreement, which improves when the number $M$ of synthetic sequences increases, and, in the case of the diagonal elements, with the order of the expansion in $\tilde{\kappa}$. Despite the fact that these expansions are made in the limit of small selection strength $\tilde{\kappa}$, when $\tilde{\kappa}$ is very small, the data is noisy, and agreement is thus less good that when $\tilde{\kappa}$ is somewhat larger. Interestingly, the analytical approximation Eq S27 for off-diagonal elements is very good even for intermediate and large values of $\tilde{\kappa}$, hinting that its validity extends beyond the regime of small $\tilde{\kappa}$ and beyond order 4 in $\tilde{\kappa}$. Meanwhile, for the diagonal terms, the expansion gives good results for relatively small $\tilde{\kappa}$, but agreement becomes less good as $\tilde{\kappa}$ becomes larger, in agreement with expectations for a small-$\tilde{\kappa}$ expansion.

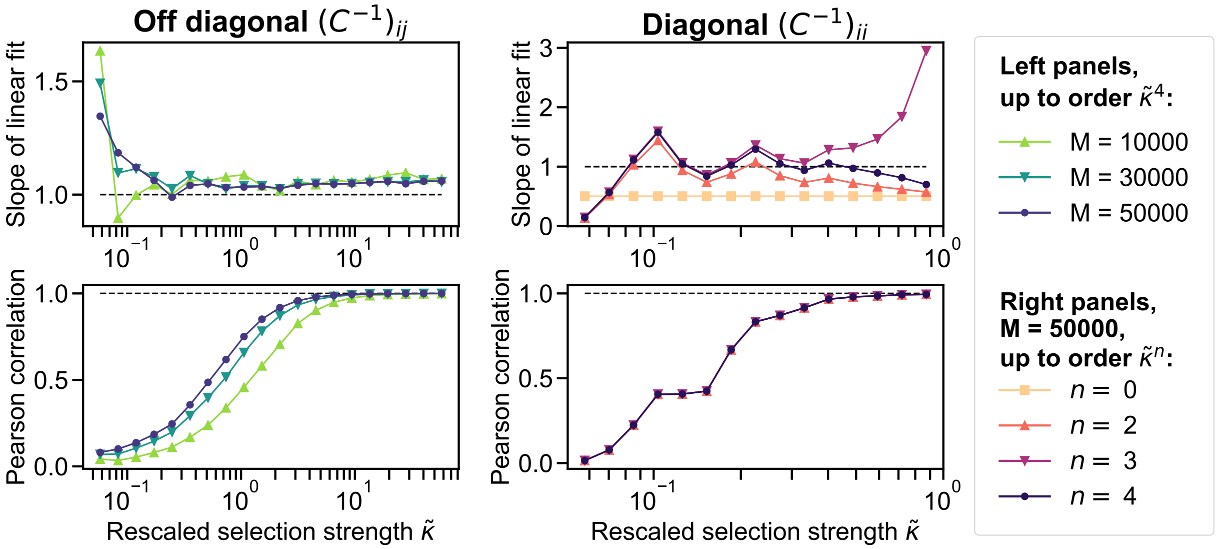


Figure I: **Inverse covariance matrix: Comparison between analytical approximation and synthetic data.** Left (resp. right) panels show the slope of the linear fit and the Pearson correlation between off-diagonal (resp. diagonal) elements of the inverse covariance matrix given by analytical formulas (Eq S26, S27) and the inverse covariance matrix computed from synthetic data. For the latter, we generate independent sequences at equilibrium as in Fig 2 but the parameter $\kappa$ is varied and $\tau^*$ is taken of order 1 (specifically, $\tau^* = 0.05\sqrt{\sum_i D_i^2} \approx 1$). In the left panels, we construct MSAs with depths $M = 10000, M = 30000, M = 50000$. In the right panels, we take $M = 50000$ sequences and compare the diagonal of the inverse covariance matrix computed from the data to the analytical formula S26 by gradually adding orders in $\tilde{\kappa}$. Note that we do not use a pseudocount ($a = 0$) here, which is acceptable given the large numbers of sequences, and allows a more direct comparison with the analytical formulas. The dashed lines represent the ideal values (slope 1 and Pearson correlation 1), which would indicate a perfect match between the data and the analytical approximation.

## 2 Impact of selection parameters on mutational effect recovery

### 2.1 Impact of the favored trait value

To what extent is recovery impacted by the favored trait value $\tau^*$, at various levels of phylogeny? To investigate this, we generate data using our minimal model for various values of $\tau^*$. Results are presented in Fig II for ICOD, covariance, SCA and conservation for three data sets with various levels of phylogeny. First, we observe that ICOD and covariance perform well at small $\tau^*$, and then recovery decays and gets poor (below null model) around $\tau^* = \sum_i D_i (\approx 196)$ before it gets somewhat better for ICOD at large $\tau^*$. SCA performs rather well at small and intermediate $\tau^*$ (except $\tau^* = 0$) but its performance deteriorates as $\tau^*$ increases. Finally, conservation performs well overall and reaches a maximum around $\tau^* = \sum_i D_i (\approx 196)$. Moreover, we observe that ICOD and conservation are more robust to phylogeny than covariance and SCA.

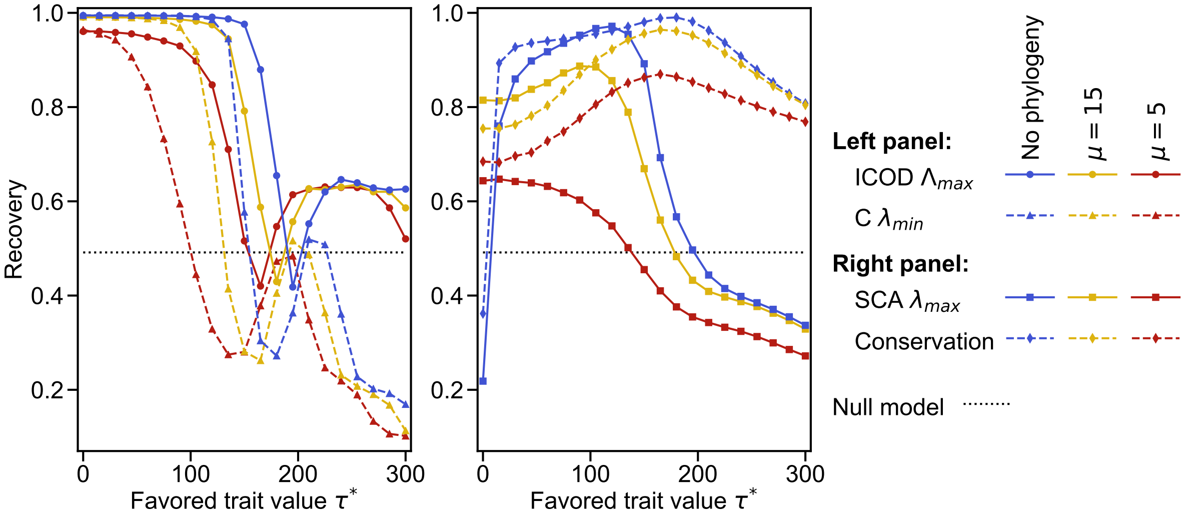


Figure II: **Impact of the favored trait value on mutational effect recovery.** The mutational effect recovery is shown as a function of the favored trait value $\tau^*$ for ICOD and covariance (left panel), and for SCA and conservation (right panel). As in Fig 3, we consider different levels of phylogeny by considering different values of $\mu$ (shown as different colors). For ICOD (resp. SCA), eigenvectors associated to the largest eigenvalue $\Lambda_{max}$ (resp. $\lambda_{max}$) are considered, while for covariance $C$, the eigenvector associated to the smallest eigenvalue $\lambda_{min}$ is considered. The null model corresponds to recovery from a random vector (see Methods, Eq 13). The data is generated exactly as in Fig 3, except that $\tau^*$ is varied. All results are averaged over 100 realisations. Results for $\tau^* \geq 0$ are shown, and those for $\tau^* < 0$ can be obtained by symmetry, see Eq 1.

In Fig III, we use symmetrized AUC (area under the receiver operating curve, see methods) to assess performance of sector site identification on the same data with the same methods. While ICOD and covariance display similar behaviours with symmetrized AUC as with recovery, SCA and conservation reach better performance with symmetrized AUC, especially for large $\tau^*$ or with little phylogeny. Indeed, conservation identifies sites associated to the sector as highly conserved, but does not recover mutational effect values, and SCA may inherit this property from conservation. However, SCA is quite hindered by phylogeny at small $\tau^*$, which limits the interest of the contribution of conservation.

Since we showed that the other end of the ICOD spectrum, corresponding to small eigenvalues, also contains information on the sector, we also compute recovery and symmetrized AUC from that end of the spectrum for ICOD, covariance and SCA, see Fig IV and V. Those results confirm that the smallest ICOD eigenvalue $\Lambda_{min}$ contains some information on the sector, especially at large values of $\tau^*$. Meanwhile, results for covariance and SCA are poor for that end of the spectrum.

Fig II, III, IV and V feature particular behaviours around $\tau^* = 190$. We remark that this value of $\tau^*$ is close to $\sum_i D_i \approx 196$, which is the value taken by the trait $\tau$ when all sites have state $+1$. Given that sector sites have much stronger mutational effects than others, reaching this value of the trait essentially

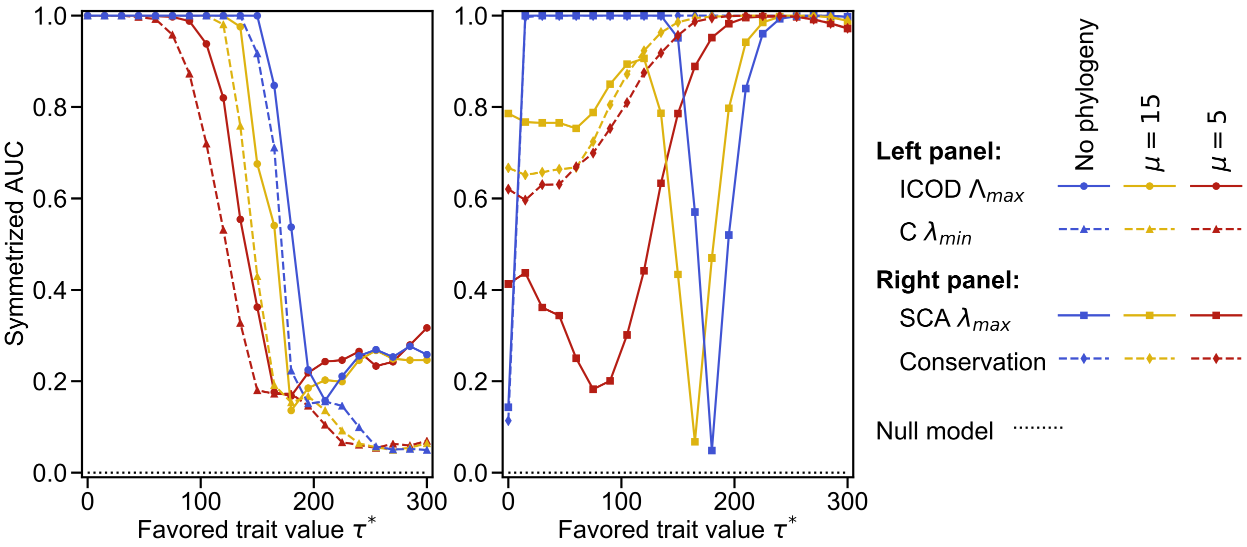


Figure III: **Impact of the favored trait value on symmetrized AUC.** Same as in Fig II, but instead of using recovery, we consider the symmetrized AUC between the eigenvectors and the mutational effects.

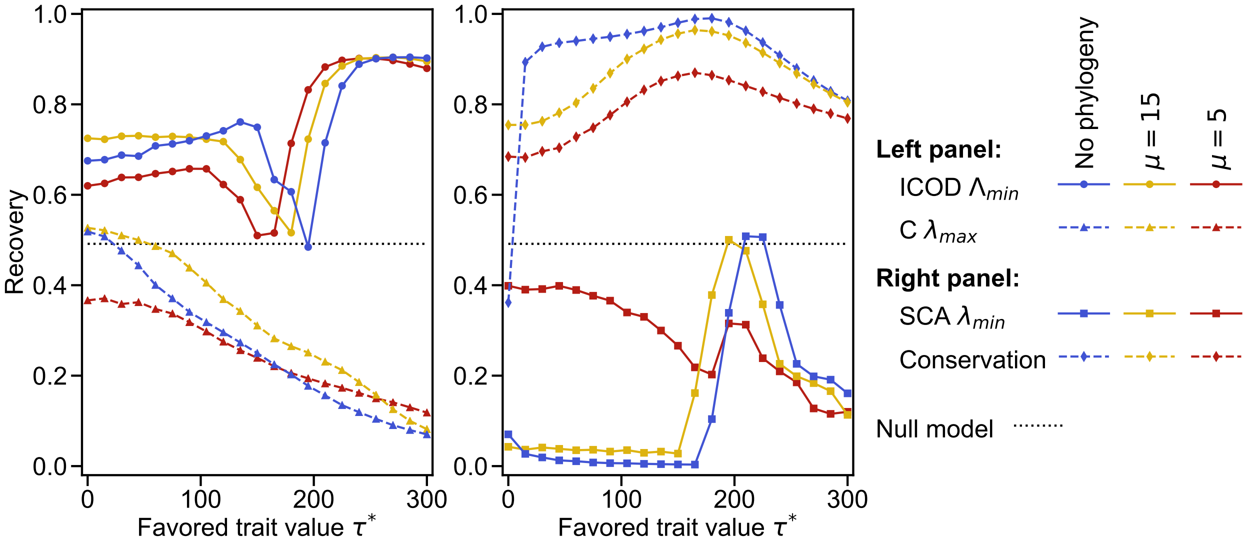


Figure IV: **Impact of the favored trait value on mutational effect recovery at the opposite end of the spectrum.** Same as in Fig II, but focusing on eigenvectors at the opposite end of the spectrum. For ICOD (resp. SCA), eigenvectors associated to the smallest eigenvalue $\Lambda_{min}$ and (resp. $\lambda_{min}$) are used, while for covariance, the eigenvector associated to the largest eigenvalue $\lambda_{max}$ is used. Conservation results (see Fig II) are reproduced here for comparison purposes.

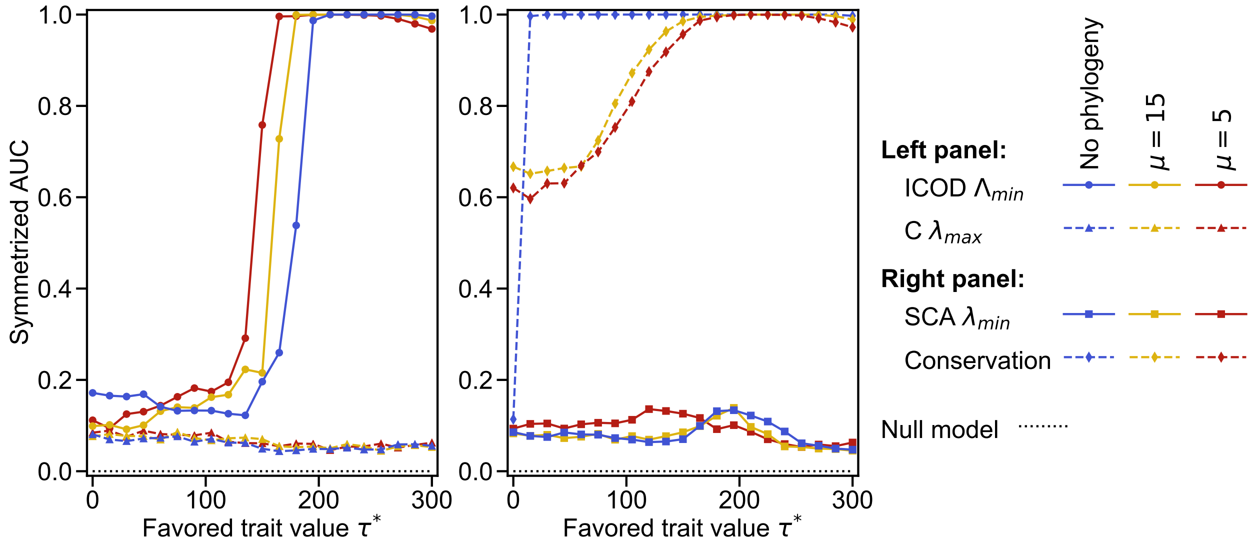


Figure V: **Impact of the favored trait value on symmetrized AUC at the opposite end of the spectrum.** Same as in Fig IV, but instead of using recovery, we consider the symmetrized AUC between the eigenvectors and the mutational effects.

requires all sector sites have state +1, which makes them strongly conserved. Accordingly, the performance of conservation in Fig II peaks around $\tau^* = 190$. Indeed, for larger $\tau^*$, even non-sector sites have to be conserved, including those with negative mutational effect, impairing recovery but not symmetrized AUC (as sector sites remain the most conserved ones), see Fig III.

The ICOD spectrum and the corresponding eigenvectors are analyzed in more detail in Fig VI for different values of $\tau^*$. In the spectrum, the number of positive outliers increases when $\tau^*$ is increased, and these eigenvalues become very large, while the number of negative outliers reduces to one. For large $\tau^*$, sector sites are extremely conserved, and each of them gives rise to a direction of very low variance [3], which results in very large eigenvalues of the ICOD matrix due to the matrix inversion step. In this regime, we do not observe such a split of sector signal at the opposite end of the spectrum (most negative eigenvalue), which contains information on the sector as well (see above). Accordingly, the eigenvector associated to the largest eigenvalue $\Lambda_{max}$ of the ICOD matrix recovers the mutational effect vector $\vec{D}$ very well for moderate $\tau^*$, but possesses large components only on a few of the sector sites for larger $\tau^*$. Meanwhile, the eigenvector associated to $\Lambda_{min}$ has large components on all sector sites for larger $\tau^*$, which is not the case for smaller $\tau^*$.

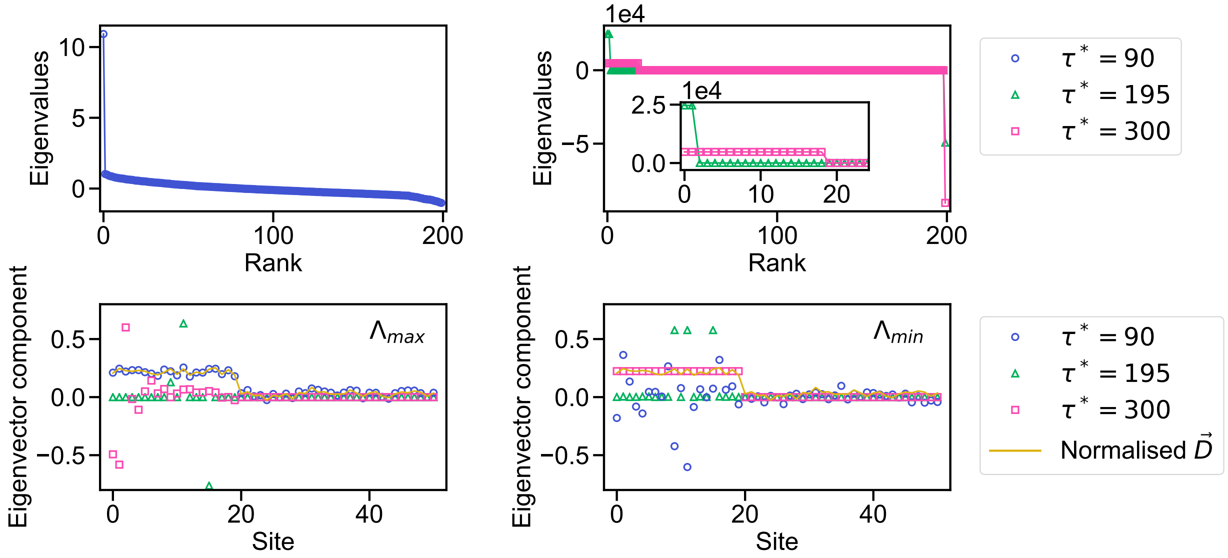


Figure VI: **Impact of large favored trait values on ICOD spectrum and eigenvectors.** Top panels show the eigenvalues of the ICOD matrix at different values of favored trait value $\tau^*$ (left: $\tau^* = 90$; right: $\tau^* = 195$ and $\tau^* = 300$). Inset in the upper right panel: zoom on the 20 first eigenvalues. Bottom panels show the normalised eigenvectors associated to the largest eigenvalue ($\Lambda_{max}$) on the left and smallest eigenvalue ($\Lambda_{min}$) on the right panel, for the same three values of $\tau^*$ as in the top panels. In the bottom panels, we only show the first 50 sites of the eigenvectors, which comprise the 20 sites associated to the sector and 30 non-sector sites. The vector of mutational effects $\vec{D}$ is normalised and shown for comparison with the eigenvectors. Data is generated as in the equilibrium (no phylogeny) case in Fig 3, but using different values of $\tau^*$.

## 2.2 Impact of selection strength

How do selection strength and phylogeny combine and impact the performance of mutational effect recovery? To address this, we generate data using our minimal model for various values of selection strength $\kappa$. Results are shown in Fig VII for ICOD and covariance, with two values of $\tau^*$ and various levels of phylogeny.

For $\tau^* = 90$, mutational effect recovery by ICOD and covariance is quite robust to selection strength $\kappa$, apart from the very weak selection case where recovery is low because sequences are noisy. Furthermore, covariance is more negatively impacted by phylogeny than ICOD across values of $\kappa$.

For $\tau^* = 140$, we observe that recovery tends to decrease when $\kappa$ increases (ignoring the very weak selection regime). However, this only happens with substantial phylogeny ($\mu = 5$) for ICOD, while this effect is always present for covariance, but becomes stronger when phylogeny is stronger. In this case, phylogeny makes recovery by covariance poor, presumably because the combination of strong phylogeny and large $\tau^*$ yields strong conservation. Even in the absence of phylogeny, recovery by covariance decreases as selection strength increases for $\tau^* = 140$. This can also be attributed to increasing conservation, to which ICOD is more robust. Consistently, Fig VIII shows that conservation yields good recovery for $\tau^* = 140$, which does not deteriorate too much due to phylogeny. The latter figure also shows that recovery by SCA is impaired by strong selection and phylogeny.

Overall, ICOD and conservation are the most robust methods to stronger selection and phylogeny.

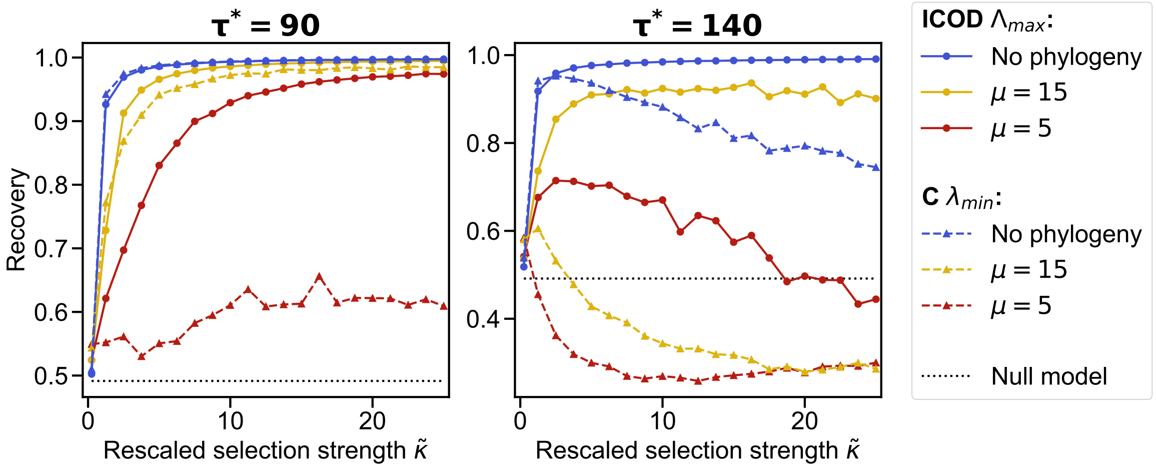


Figure VII: **Impact of selection strength on mutational effect recovery.** Recovery by covariance and ICOD is shown as a function of the rescaled selection strength $\tilde{\kappa} = \kappa \sum_i D_i^2$ for three data sets generated with different levels of phylogeny, and with favored trait value $\tau^* = 90$ (left) or $\tau^* = 140$ (right). For ICOD, the eigenvector associated to the largest eigenvalue $\Lambda_{max}$ is considered, while for covariance $C$, the eigenvector corresponding to the smallest eigenvalue $\lambda_{min}$ is considered. The data is generated as in Fig 3, except that $\kappa$ is varied. All results are averaged over 100 realisations.

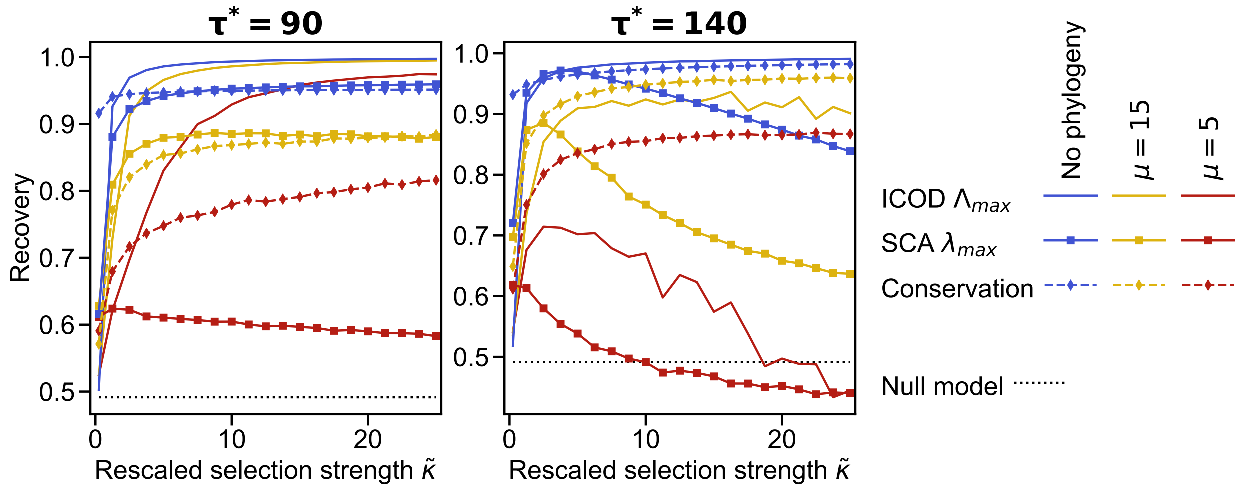


Figure VIII: **Impact of selection strength on mutational effect recovery for SCA and conservation.** Same as in Fig VII but using SCA and conservation. For SCA, the eigenvector associated to the largest eigenvalue ($\lambda_{max}$) is used. ICOD results (see Fig VII) are reproduced here for comparison purposes.

# References


1. Chaikin PM, Lubensky TC. Principles of condensed matter physics. Cambridge University Press; 1995.
2. Sjöstrand J. Singularités analytiques microlocales. Astérisque. 1982;95:III–166.
3. Wang SW, Bitbol AF, Wingreen N. Revealing evolutionary constraints on proteins through sequence analysis. PLoS Comput Biol. 2019;15(4):e1007010.
4. Maillard A, Foini L, Castellanos AL, Krzakala F, Mézard M, Zdeborová L. High-temperature expansions and message passing algorithms. Journal of Statistical Mechanics: Theory and Experiment. 2019;2019(11):113301.
