# Supplementary figures and images for: Impact of phylogeny on the inference of functional sectors from protein sequence data
Source: PLoS Comput Biol. 2024 Sep 23;20(9):e1012091. doi: 10.1371/journal.pcbi.1012091 (PMC11449291; doi:10.1371/journal.pcbi.1012091)

$$\tilde{C}_{ij}^{-1}$$

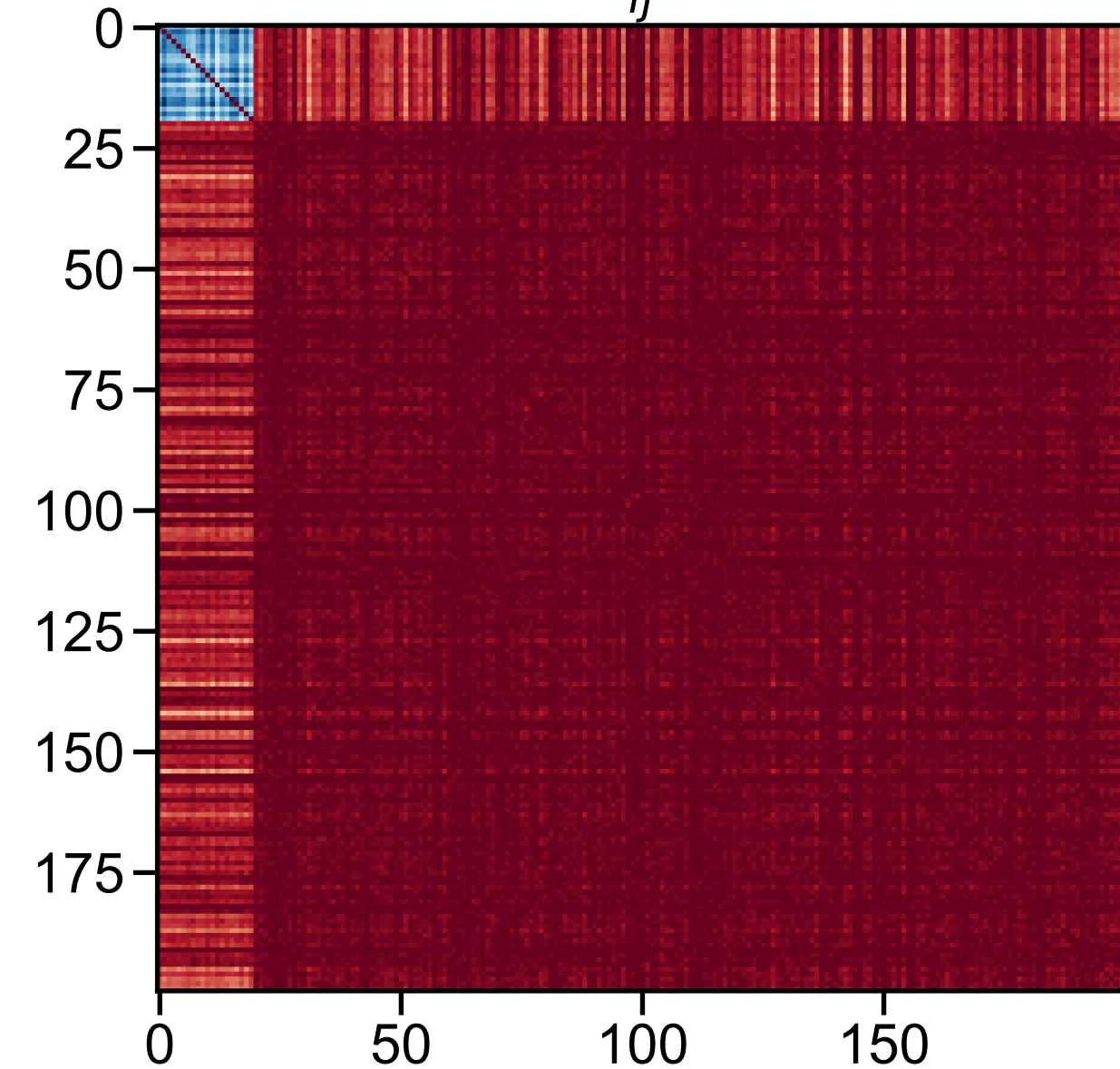

Block diagonal approximation of  $\tilde{C}_{ij}^{-1}$

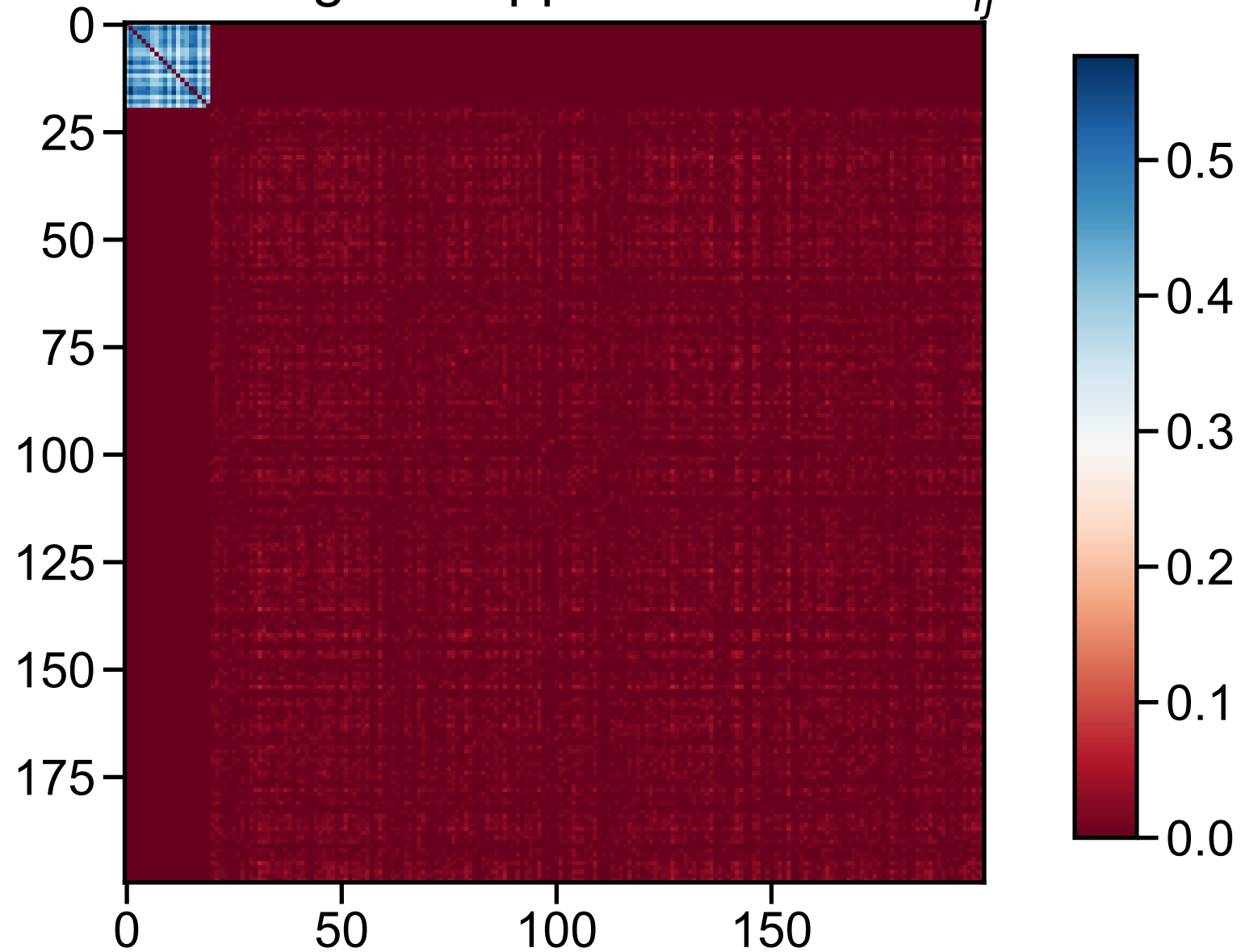

Supplement: S1 Fig — The left panel shows the ICOD matrix computed on data generated independently at equilibrium (14000 sequences). Parameters are the same as for the equilibrium (‘No phylogeny’) data set in Fig 3. Recall that LS = 20 sector sites out of L = 200 total sites. The first 20 × 20 diagonal block (mainly blue) is associated to the sector, while the second one, of size 180 × 180, is associated to non-sector sites (i.e. sites with small mutational effects). The right panel shows the block diagonal approximation of the ICOD matrix shown in the left panel. Here, the matrix elements that do not belong to either of the two diagonal blocks are set to 0. Meanwhile, elements of these diagonal blocks are the same as in the ICOD matrix. (PDF) [file pcbi.1012091.s002.pdf]

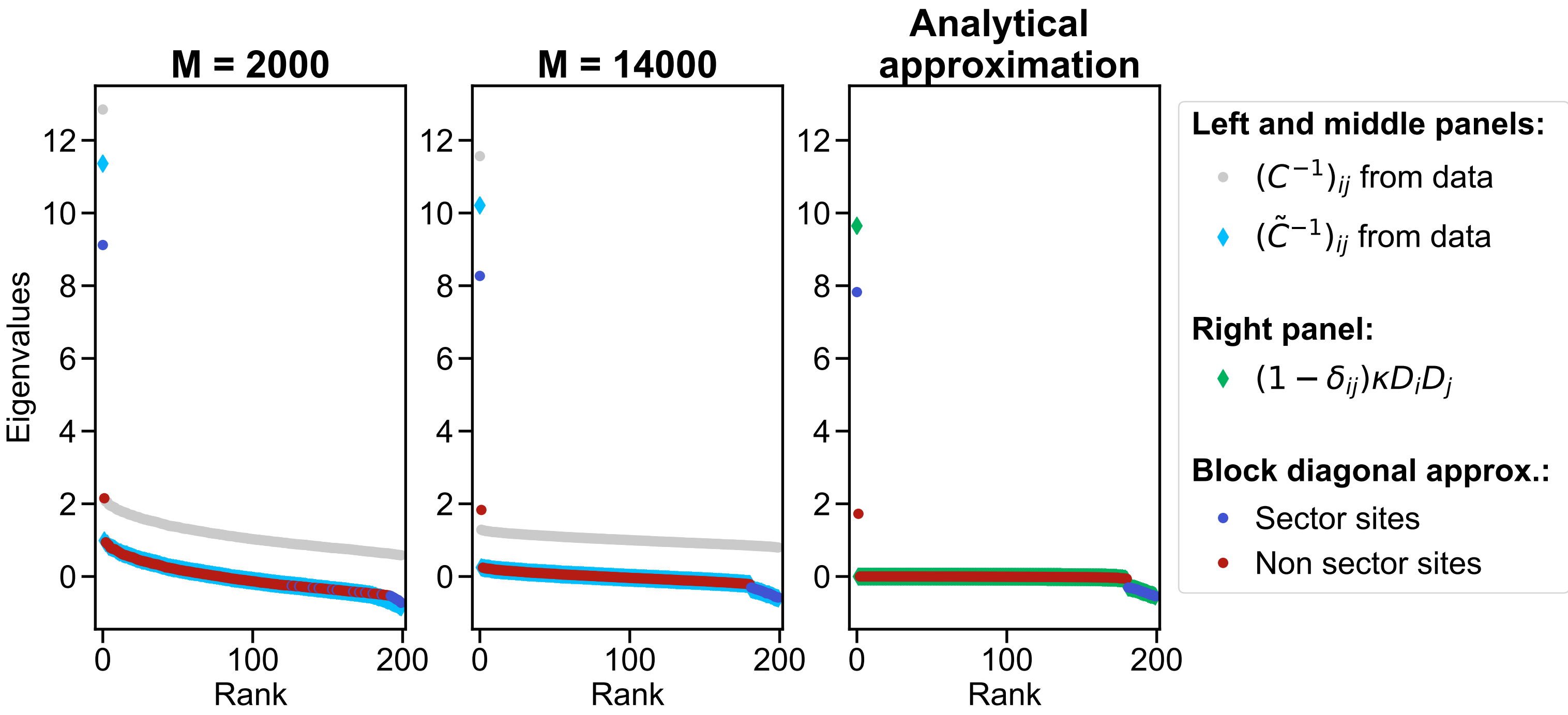

Supplement: S2 Fig — Same as in Fig 2, except that the vector D→ of mutational effects comprises both negative and positive components. Specifically, we took the same D→ as in Fig 2, but we multiplied the 10 first components (corresponding to half of the sector) by −1. (PDF) [file pcbi.1012091.s003.pdf]

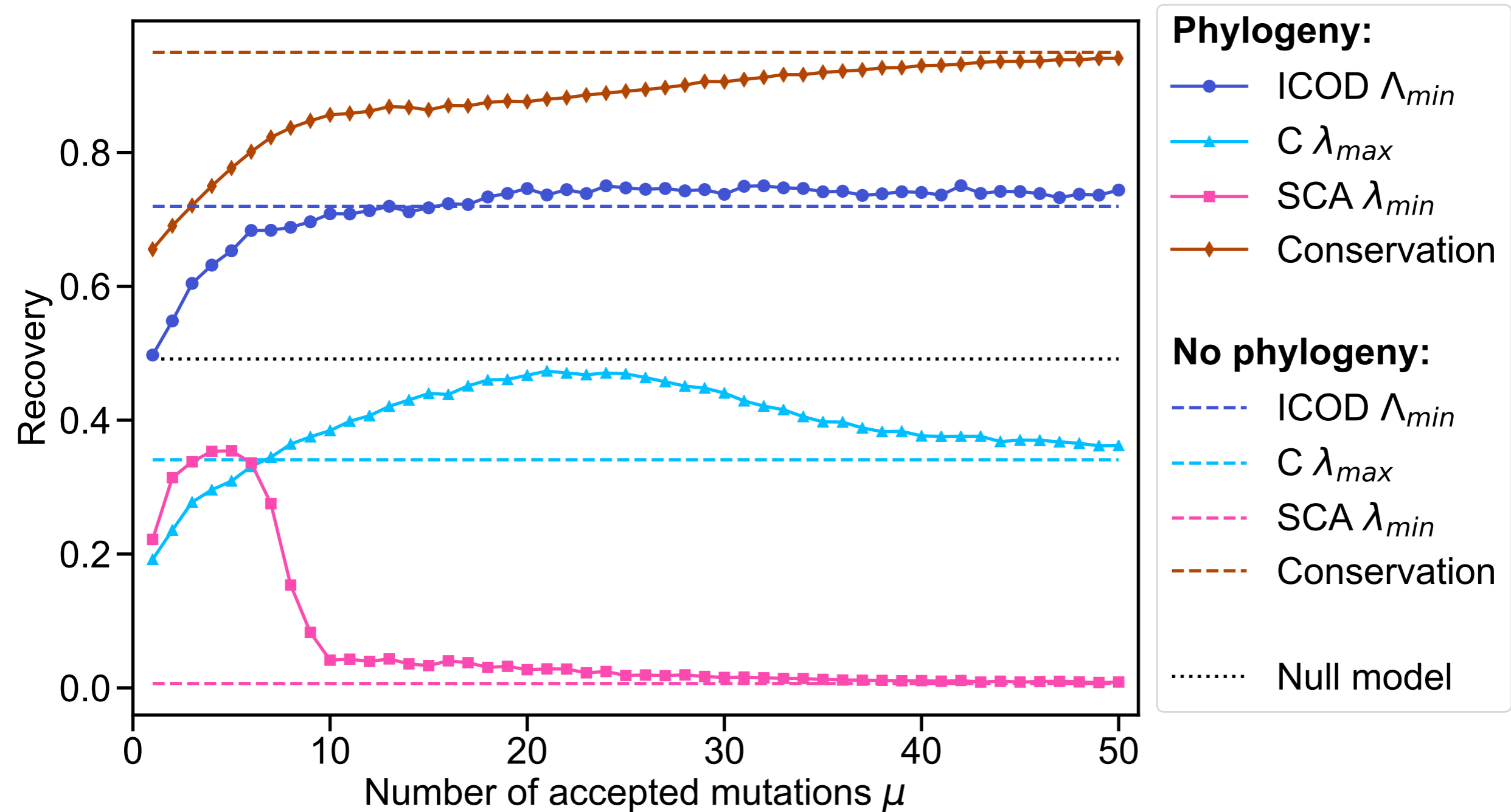

Supplement: S3 Fig — Same as in Fig 4, but using the eigenvectors associated to the eigenvalues at the opposite end of the spectrum. For ICOD (resp. SCA), eigenvectors associated to the smallest eigenvalue Λmin (resp. λmin) are considered. For covariance C, the eigenvector associated to the largest eigenvalue λmax is considered. (PDF) [file pcbi.1012091.s004.pdf]

No phylogeny

ICOD

Covariance

SCA

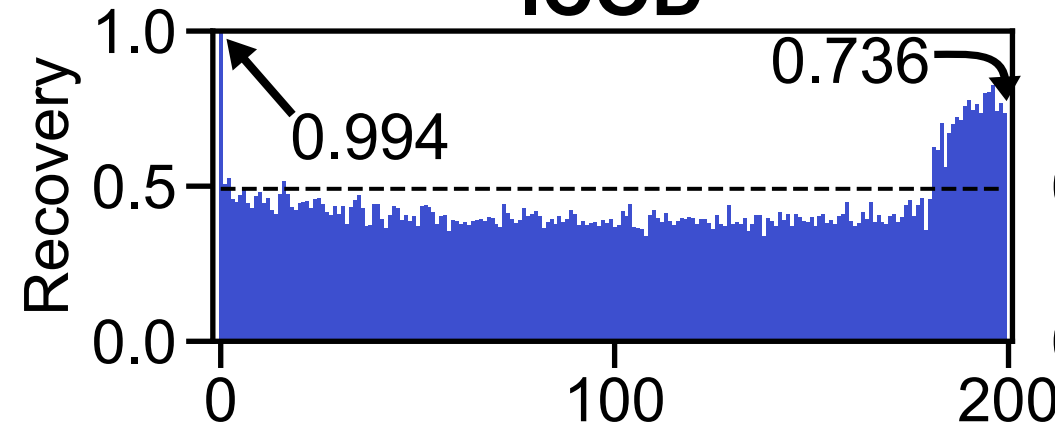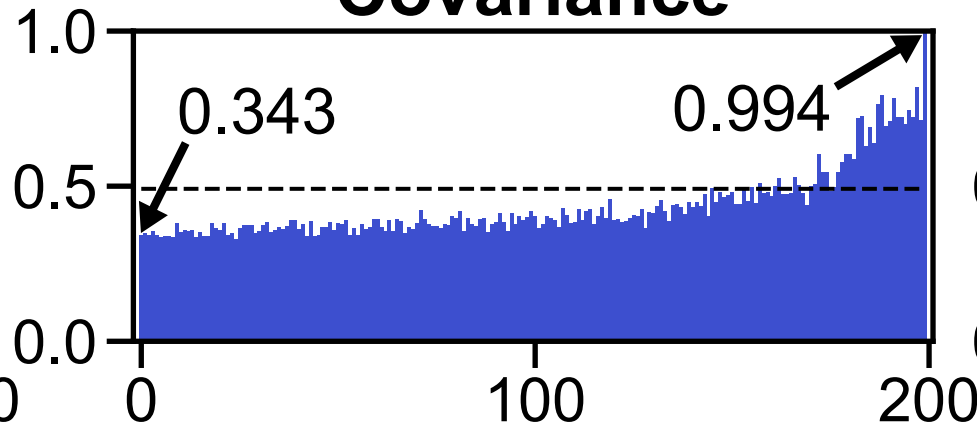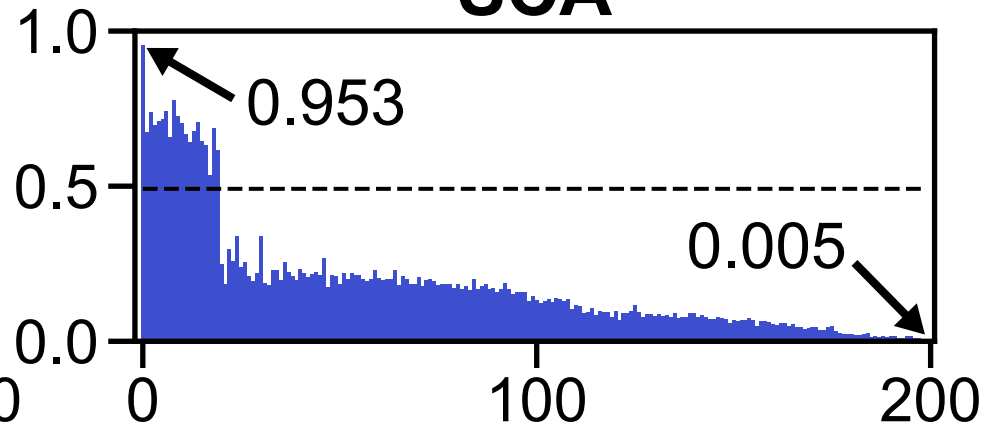

$\mu = 15$

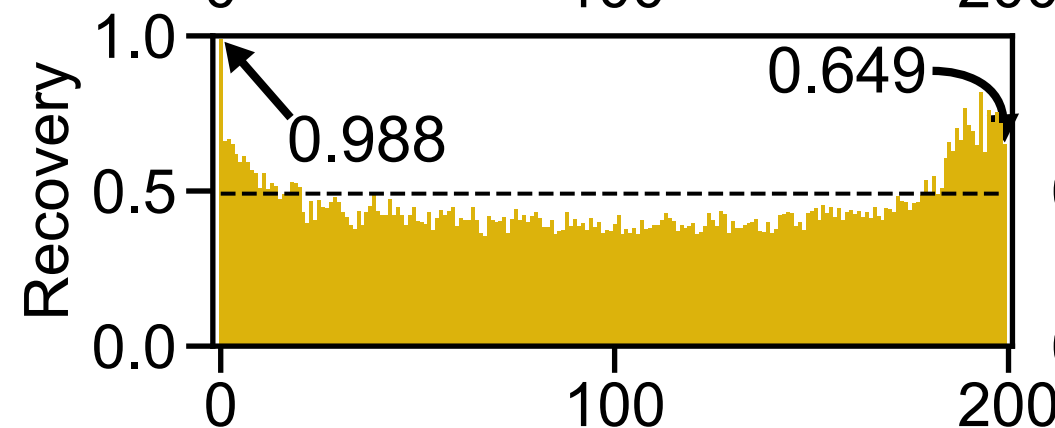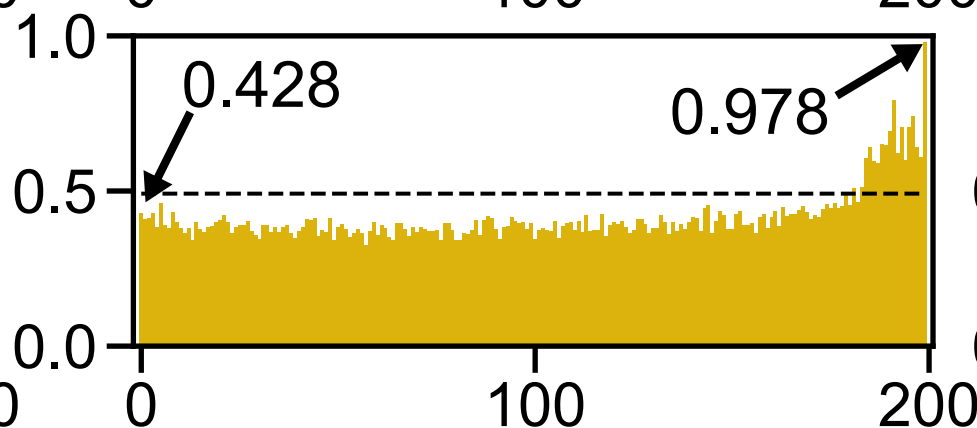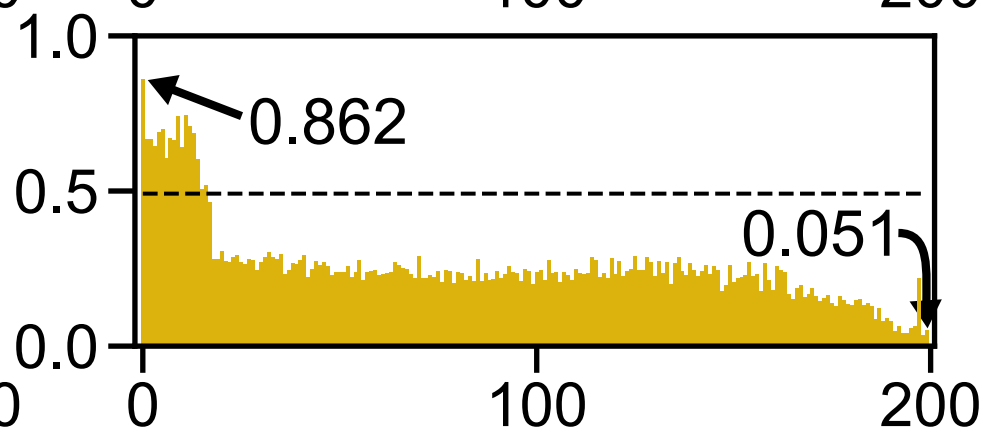

$\mu = 5$

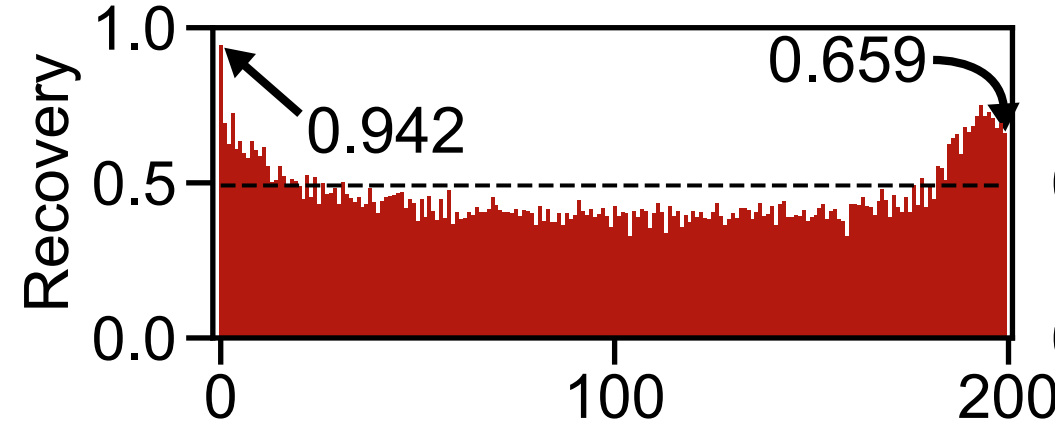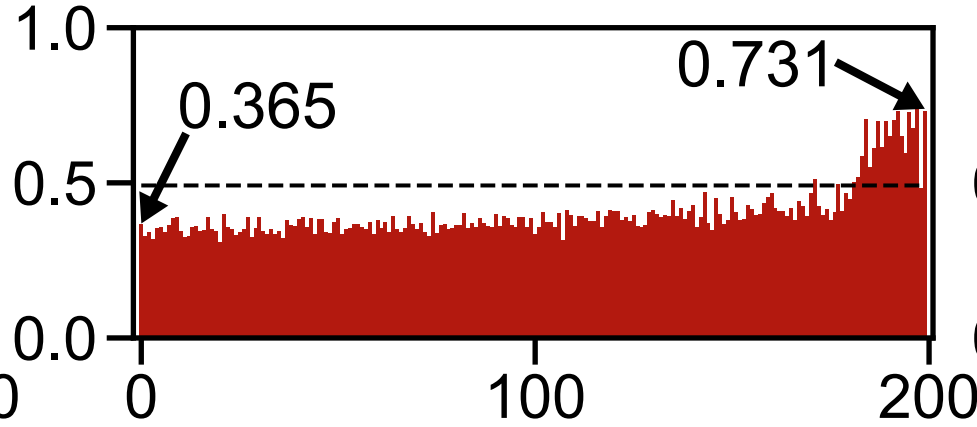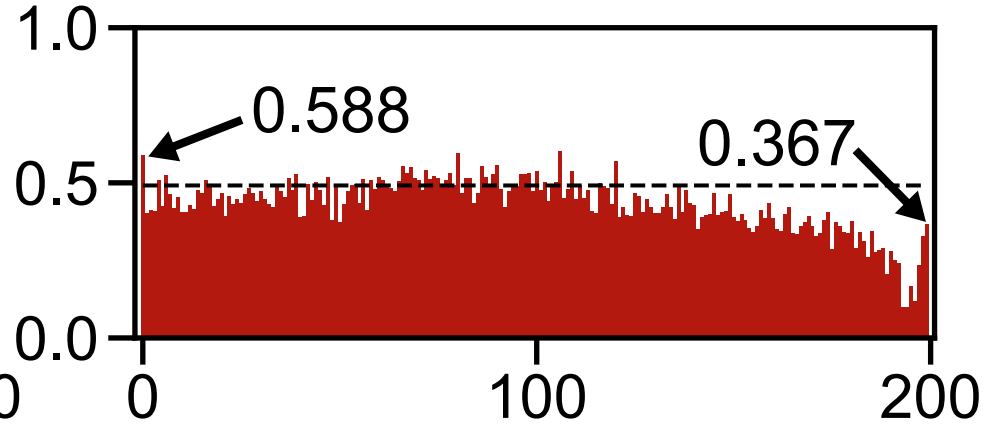

Rank

Rank

Rank

Supplement: S4 Fig — The mutational effect recovery is shown for three methods: ICOD, Covariance and SCA, using each eigenvectors in the spectrum of the matrix involved in the method. The dashed line shows the chance expectation for the recovery (see Eq 13). Recovery by the eigenvectors associated to the largest and smallest eigenvalues are indicated for each method. Throughout, three data sets are considered, which differ by the amount of phylogeny (No phylogeny, μ = 15, μ = 5). The data is generated as in Fig 3. (PDF) [file pcbi.1012091.s005.pdf]

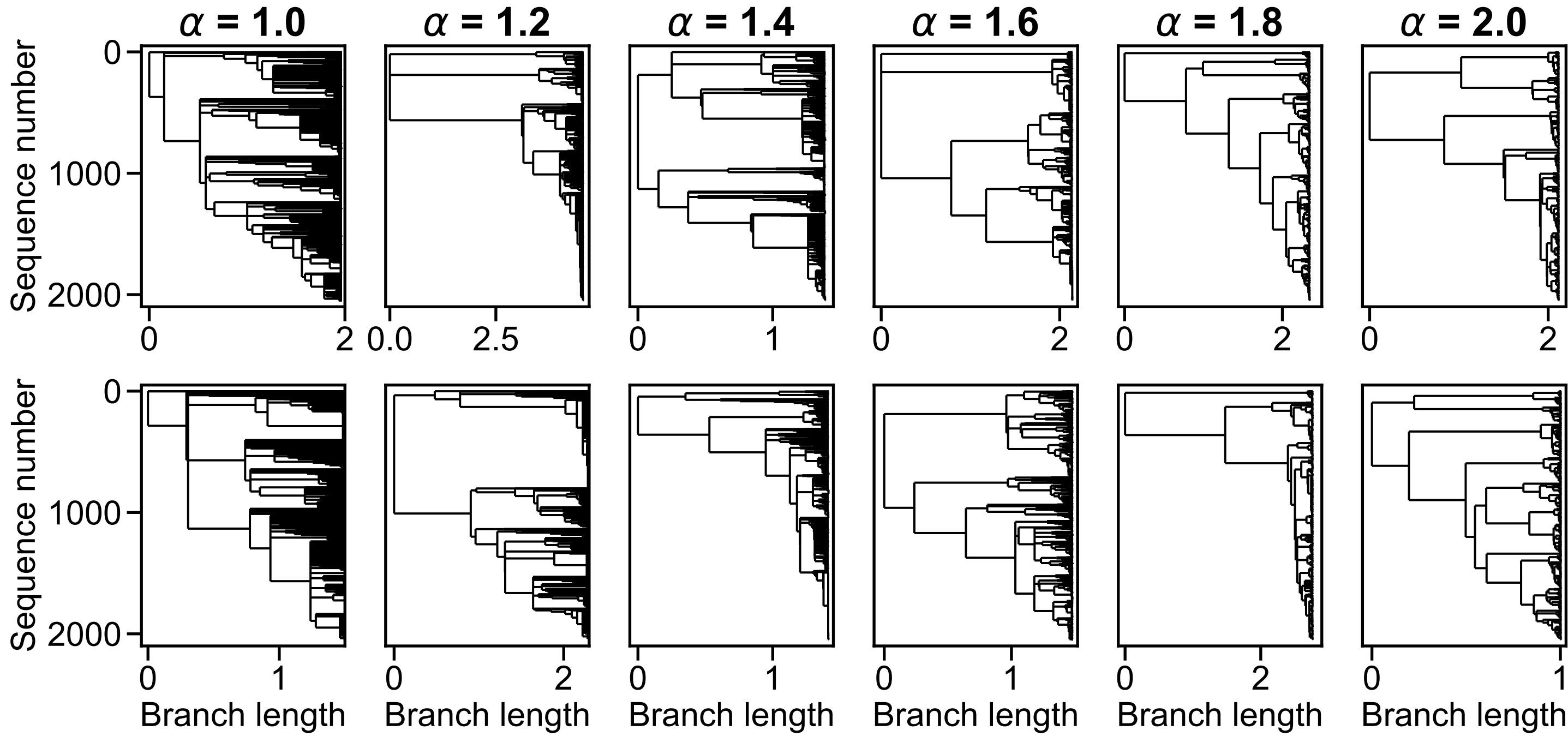

Supplement: S5 Fig — Beta-coalescent trees are generated using the code associated to Ref. [49], which is available on GitHub (https://github.com/rneher/betatree). Each column corresponds to a specific value of the parameter α which characterizes the Beta-coalescent tree. For each value of α, we sampled two trees, represented in the two rows of the figure. Note that α = 1 corresponds to the Bolthausen-Sznitman coalescent, while α = 2 corresponds to the Kingman coalescent. (PDF) [file pcbi.1012091.s006.pdf]

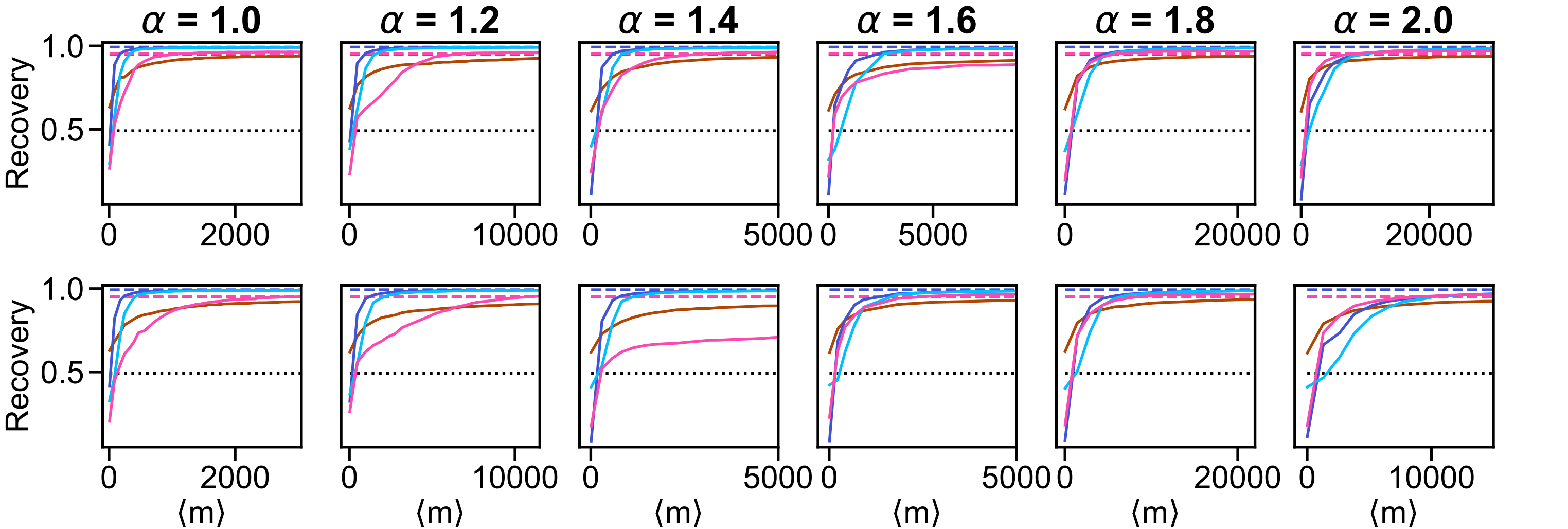

Supplement: S6 Fig — Same analysis as in Fig 4, but using data generated along the Beta-coalescent trees shown in S5 Fig instead of a perfect binary tree. Each panel here is associated to the tree shown in the corresponding panel of S5 Fig. Mutational effect recovery is shown for various methods versus the average number 〈m〉 of accepted mutations between the ancestor and a leaf. For synthetic data generation, the number of accepted mutations along each branch is drawn from a Poisson law with mean equal to a multiplicative constant times the branch length shown in S5 Fig. The multiplicative constant (which is the same for all branches in a tree) is then varied to tune the amount of phylogeny in the data, yielding various values of 〈m〉. All curves are averaged over 100 realisations of data generation along the same tree. We checked that the asymptotic values obtained without phylogeny (dashed lines) are reached for large 〈m〉. (PDF) [file pcbi.1012091.s007.pdf]

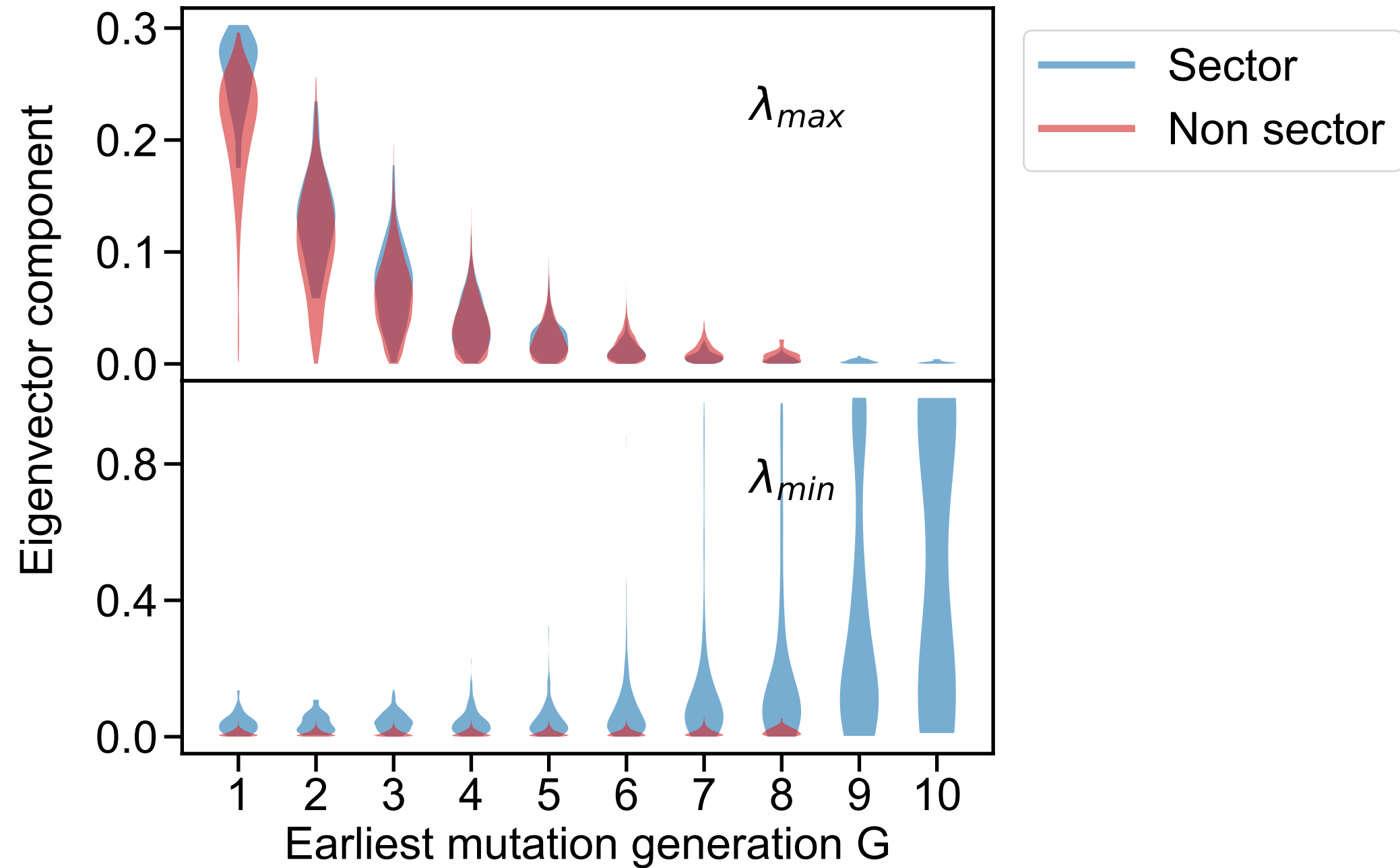

Supplement: S7 Fig — Same as in Fig 5, restricting to the data set with μ = 5 and to the covariance method. Top panel: eigenvector associated to the largest eigenvalue λmax of the covariance matrix. Bottom panel: results for the eigenvector associated to the smallest eigenvalue λmin (see Fig 5) are reproduced here for comparison purposes. (PDF) [file pcbi.1012091.s008.pdf]

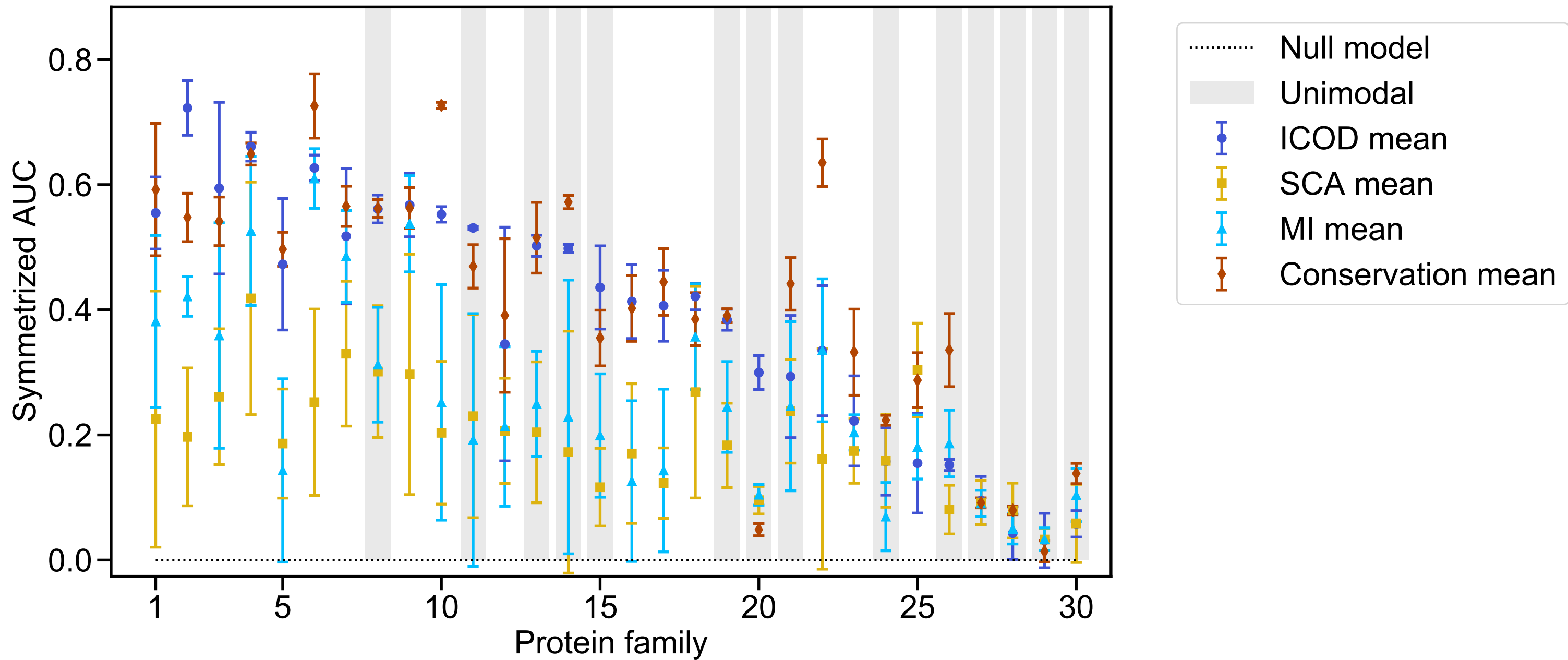

Supplement: S8 Fig — Same as in Fig 6, but using the mean of the performance, i.e. mean of the symmetrized AUCs, over all phylogenetic cutoffs considered. The error bars represent the standard deviation over phylogenetic cutoffs. Here, the average of the symmetrized AUC over all families is 0.42 for conservation, 0.39 for ICOD, 0.25 for MI and 0.19 for SCA. The mapping between protein family number and name is given in S1 Table. (PDF) [file pcbi.1012091.s009.pdf]

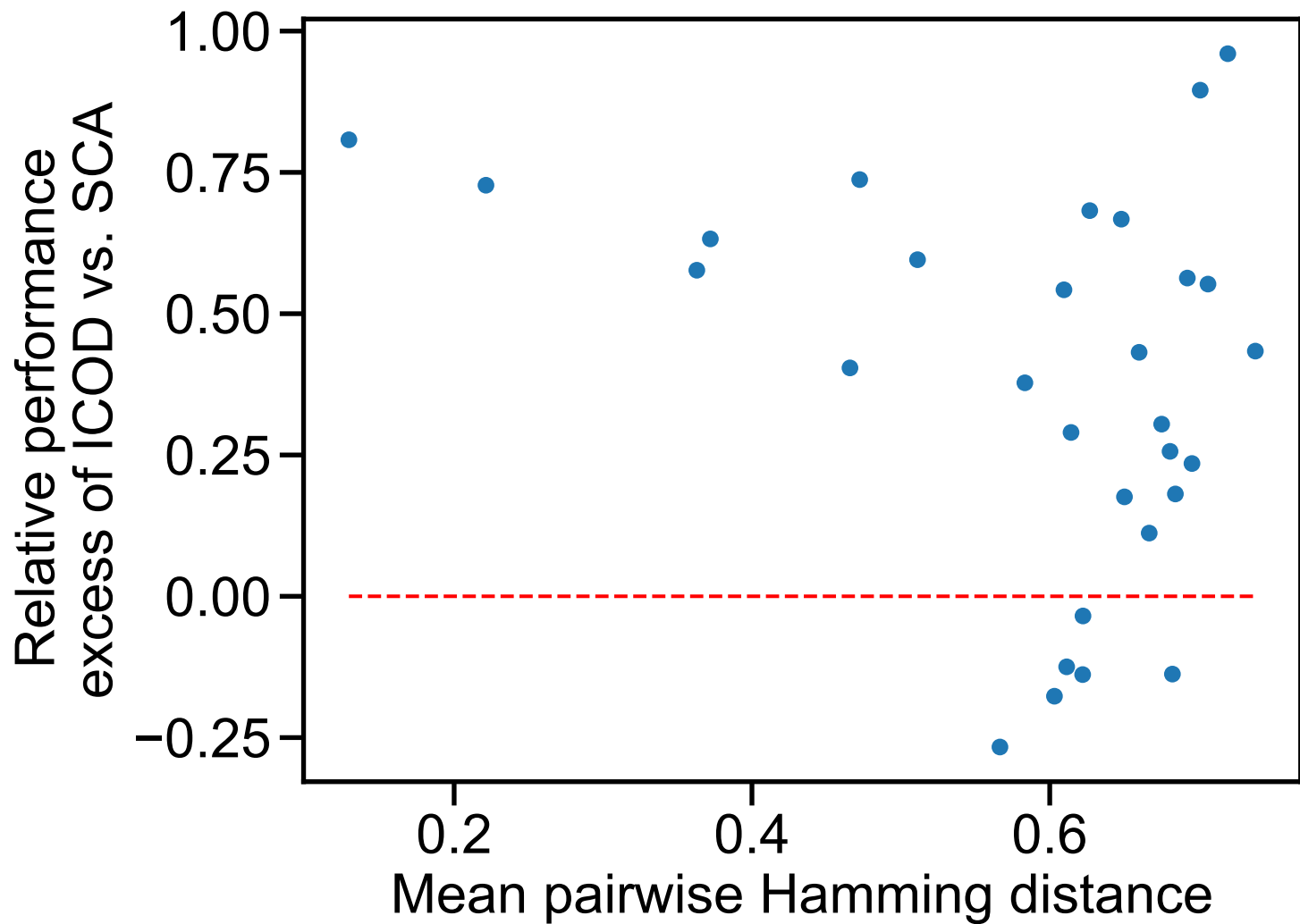

Supplement: S10 Fig — The relative difference between the symmetrized AUC score S for ICOD and SCA (i.e., (SICOD − SSCA)/SICOD) is plotted versus the mean pairwise Hamming distance in the MSA, for the prediction of sites with large mutational effects in 30 different protein families. The red dashed line separates the cases where ICOD is best (positive values) from those where SCA is best (negative values). Performances measured as symmetrized AUC values are the same as in Fig 6, and protein families are listed in S1 Table. For each family, the mean pairwise Hamming distance is computed for the MSA associated to the maximum phylogenetic cutoff Cmax considered, thus reflecting the largest diversity in the family, see S2 Table. Phylogenetic cutoffs are defined and their values are given in the paragraph “MSA construction” of the Methods section. (PDF) [file pcbi.1012091.s011.pdf]

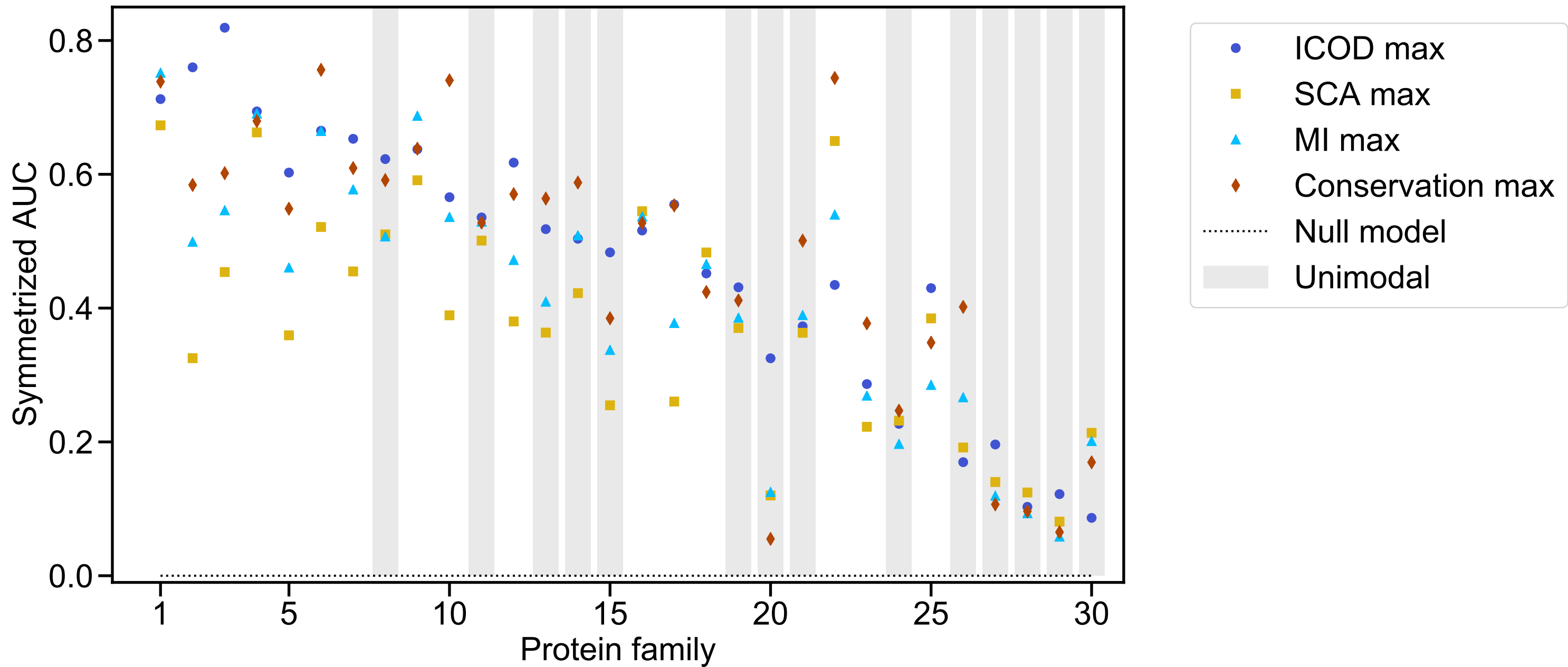

Supplement: S11 Fig — Same as in Fig 6, but focusing on the MSA phylogenetic cutoffs that maximize the symmetrized AUC. The mapping between protein family number and name is given in S1 Table. (PDF) [file pcbi.1012091.s012.pdf]

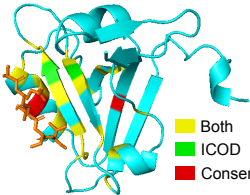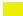

Both

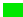

ICOD

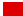

Conservation

Supplement: S12 Fig — The structure of the PDZ domain is represented in cyan and the CRIPT molecule is shown in orange. True positive sites of the sector found both by ICOD and by conservation are colored in yellow, while TP sites found by ICOD only are shown in green and TP sites found by conservation only are shown in red. The PDB identifier of this structure is 1BE9. (PDF) [file pcbi.1012091.s013.pdf]

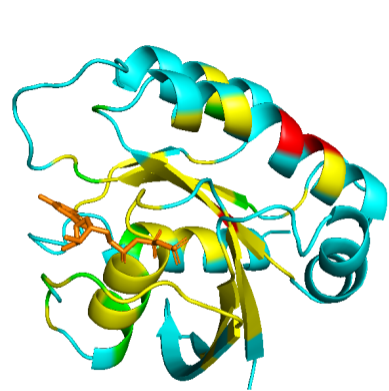

(a) Ras and GNP structure

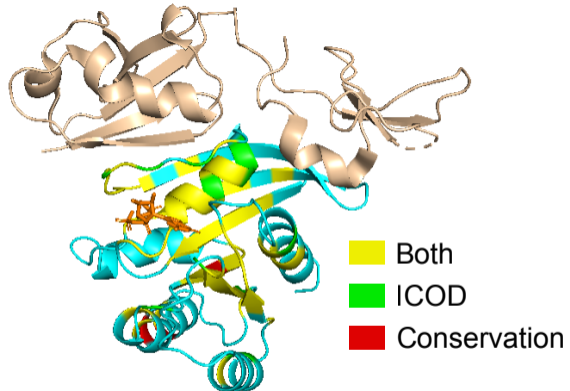

(b) GNP-Ras-Raf complex

Supplement: S13 Fig — In both panels, the structure of Ras is shown in cyan. True positive sites of the sector found both by ICOD and by conservation are colored in yellow, while TP sites found by ICOD only are shown in green and TP sites found by conservation only are shown in red. (a) Structure of Ras in interaction with the GNP molecule (colored in orange). PDB identifier: 5P21. (b) Structure of Ras in complex with the RBD and CRD domains of Raf (colored in wheat). The GNP molecule is again colored in orange. PDB identifier: 6XI7. (PDF) [file pcbi.1012091.s014.pdf]
