# Supplementary material for: Impact of phylogeny on the inference of functional sectors from protein sequence data
Source: PLoS Comput Biol. 2024 Sep 23;20(9):e1012091. doi: 10.1371/journal.pcbi.1012091 (PMC11449291; doi:10.1371/journal.pcbi.1012091)
Supplement: S9 Fig — Histograms of the minimum DMS scores (see Methods) are shown in six example cases. Top panels: three examples of scores with bimodal shape (or more complex shape—middle panel, see Methods). Bottom panels: three examples of scores with unimodal shape. In each case, bimodal Gaussian (in top panels) or Gaussian fits (in bottom panels) are shown together with their respective cutoffs, which correspond either to the location of the minimum value between peaks for bimodal fits, or to the mean for Gaussian fits (see Methods). (PDF) [file pcbi.1012091.s010.pdf]

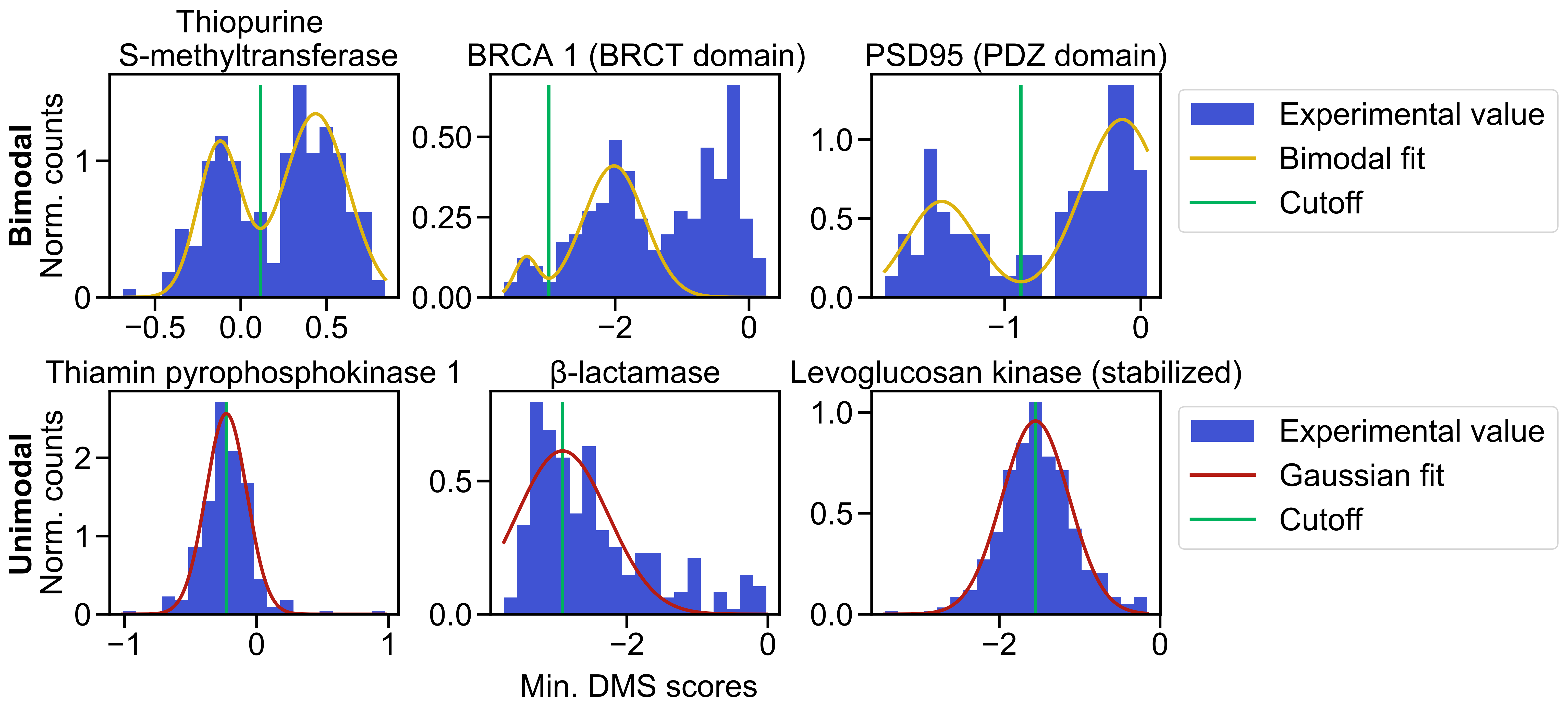
Thiopurine S-methyltransferase
BRCA 1 (BRCT domain)
PSD95 (PDZ domain)
Bimodal
Norm. counts
Experimental value
Bimodal fit
Cutoff
Thiamin pyrophosphokinase 1
β-lactamase
Levoglucosan kinase (stabilized)
Unimodal
Norm. counts
Experimental value
Gaussian fit
Cutoff
Min. DMS scores
