# Supplementary material for: Impact of phylogeny on the inference of functional sectors from protein sequence data
Source: PLoS Comput Biol. 2024 Sep 23;20(9):e1012091. doi: 10.1371/journal.pcbi.1012091 (PMC11449291; doi:10.1371/journal.pcbi.1012091)
Supplement: S1 Table — The first two columns present the name of natural protein families that are considered and the corresponding label used in Fig 6 and S11 Fig. Next, the ‘DMS’ column indicates the shape of the DMS data, 1 for unimodal and 2 for bimodal. The symmetrized AUC columns correspond to the results shown in Fig 6—conservation is abbreviated by ‘Cons.’. The best score among the methods is highlighted in bold for each family. Finally, the number of true positive sites found both by ICOD and by conservation (‘Both’), by ICOD only (‘ICOD’) and by conservation only (‘Cons.’) are given. The last columns provide the size (or length) LS of the sector and the protein length L. (PDF) [file pcbi.1012091.s015.pdf]

Name	Label	DMS	Symmetrized AUC				True positives			L_S	L
			ICOD	Cons.	SCA	MI	Both	ICOD	Cons.		
GAL4 (DNA-binding domain)	1	2	0.76	**0.77**	0.57	0.37	29	1	2	34	43
Translation initiation factor IF1	2	2	**0.76**	0.56	0.31	0.49	12	5	2	23	69
PABP singles (RRM domain)	3	2	**0.73**	0.62	0.33	0.69	15	3	1	25	71
Kanamycin kinase APH(3')-II	4	2	**0.70**	0.68	0.62	0.67	119	12	9	158	244
HRas	5	2	**0.68**	0.54	0.30	0.26	55	11	3	83	158
HSP90 (ATPase domain)	6	2	0.66	**0.76**	0.41	0.69	67	4	7	94	216
PSD95 (PDZ domain)	7	2	0.60	**0.63**	0.50	0.60	18	2	2	28	77
β-lactamase	8	1	**0.60**	0.59	0.42	0.45	63	12	8	107	256
BRCA 1 (RING domain)	9	2	0.57	**0.60**	0.43	0.59	13	1	1	23	70
HIV env protein (BF520)	10	2	0.55	**0.73**	0.20	0.48	242	7	27	295	356
Influenza polymerase PA subunit	11	1	**0.53**	0.46	0.10	0.36	209	35	22	346	683
DNA methylase HaeIII	12	2	**0.53**	0.48	0.30	0.31	129	14	4	179	306
Aliphatic amide hydrolase	13	1	0.51	**0.53**	0.13	0.29	100	17	15	163	316
HIV env protein (BG505)	14	1	0.50	**0.57**	0.21	0.38	95	18	16	167	363
Small ubiquitin-related modifier 1	15	1	**0.48**	0.38	0.22	0.34	18	5	3	35	82
UBE4B (U-box domain)	16	2	**0.45**	0.43	0.25	0.22	34	6	5	52	76
β-glucosidase	17	2	0.44	**0.48**	0.15	0.33	219	24	29	314	441
BRCA 1 (BRCT domain)	18	2	0.42	0.41	0.35	**0.44**	1	2	1	11	208
Influenza hemagglutinin	19	1	**0.39**	**0.39**	0.23	0.29	123	18	21	241	544
Hepatitis C NS5A	20	1	**0.33**	0.05	0.09	0.14	71	4	4	80	85
SUMO-conjugating enzyme UBC9	21	1	0.32	**0.47**	0.37	0.41	34	3	5	60	136
Ubiquitin	22	2	0.31	**0.63**	0.22	0.46	28	4	8	45	70
Thiopurine S-methyltransferase	23	2	0.25	**0.37**	0.28	0.28	44	3	6	84	210
Levoglucosan kinase (stabilized)	24	1	0.22	0.24	**0.25**	0.14	91	19	15	179	361
YAP1 (WW domain)	25	2	0.20	**0.28**	0.26	0.19	10	1	1	17	30
PTEN	26	1	0.15	**0.38**	0.18	0.27	62	7	18	123	255
Mitogen-activated protein kinase 1	27	1	**0.10**	0.09	0.01	**0.10**	55	3	4	109	214
Levoglucosan kinase	28	1	**0.09**	**0.09**	**0.09**	0.03	83	18	19	187	361
Thiamin pyrophosphokinase 1	29	1	**0.06**	0.02	0.02	0.03	53	8	6	115	222
Calmodulin-1	30	1	0.05	**0.14**	0.00	0.12	25	7	9	64	137
