# Supplementary material for: Impact of phylogeny on the inference of functional sectors from protein sequence data
Source: PLoS Comput Biol. 2024 Sep 23;20(9):e1012091. doi: 10.1371/journal.pcbi.1012091 (PMC11449291; doi:10.1371/journal.pcbi.1012091)
Supplement: S2 Table — The first column gives the name of the natural families considered. The second and the third columns give the mean Hamming distance between two sequences of the MSA, respectively for the smallest and largest phylogenetic cutoffs considered (“Cmin”, “Cmax”). Phylogenetic cutoffs are defined and their values are given in the paragraph “MSA construction” of the Methods section. The average value over all families of the mean Hamming distances for “Cmin” and “Cmax” are respectively 0.19 and 0.59. The fourth and the fifth columns give the depth of the MSA, again for the two extreme cutoffs. Finally, the sixth and the seventh columns give the effective depth [7, 77] of the MSA (where the threshold of Hamming distance below which neighbouring sequences are lumped together [7, 77] is set to 0.2), again for the two extreme cutoffs. (PDF) [file pcbi.1012091.s016.pdf]

Name	Mean Hamming distance		MSA depth		Effective MSA depth	
	$C_{\min}$	$C_{\max}$	$C_{\min}$	$C_{\max}$	$C_{\min}$	$C_{\max}$
GAL4 (DNA-binding domain)	0.22	0.68	60	159996	5	33586
Translation initiation factor IF1	0.30	0.51	6691	24949	112	2283
PABP singles (RRM domain)	0.24	0.71	779	376469	17	27819
Kanamycin kinase APH(3')-II	0.10	0.67	81	5326	9	1177
HRas	0.19	0.69	2536	197323	20	17521
HSP90 (ATPase domain)	0.21	0.58	6053	59147	30	4735
PSD95 (PDZ domain)	0.14	0.65	21798	380434	16	4664
β-lactamase	0.19	0.68	1578	28469	15	5726
BRCA 1 (RING domain)	0.14	0.70	1371	152038	7	13296
HIV env protein (BF520)	0.28	0.37	71090	116165	1440	2468
Influenza polymerase PA subunit	0.04	0.13	26965	29493	2	8
DNA methylase HaeIII	0.28	0.74	238	67979	33	19487
Aliphatic amide hydrolase	0.21	0.47	2838	5293	19	109
HIV env protein (BG505)	0.27	0.36	88575	116036	1267	1640
Small ubiquitin-related modifier 1	0.14	0.61	740	8179	15	1518
UBE4B (U-box domain)	0.15	0.66	2408	38885	36	4631
β-glucosidase	0.29	0.65	1916	121829	70	12942
BRCA 1 (BRCT domain)	0.14	0.68	935	5277	10	728
Influenza hemagglutinin	0.14	0.47	19604	62216	3	24
Hepatitis C NS5A	0.18	0.22	8417	16996	3	85
SUMO-conjugating enzyme UBC9	0.24	0.68	1293	69698	34	6728
Ubiquitin	0.08	0.61	29430	87538	442	10175
Thiopurine S-methyltransferase	0.19	0.61	291	9912	8	2298
Levoglucosan kinase (stabilized)	0.22	0.62	369	35934	9	8353
YAP1 (WW domain)	0.17	0.57	2990	184514	13	3714
PTEN	0.12	0.60	1149	21804	3	940
Mitogen-activated protein kinase 1	0.11	0.70	2168	482226	16	35904
Levoglucosan kinase	0.22	0.62	370	35934	9	8363
Thiamin pyrophosphokinase 1	0.21	0.63	791	4328	10	1162
Calmodulin-1	0.20	0.72	5947	90703	180	11278
