# Supplementary material for: Impact of phylogeny on the inference of functional sectors from protein sequence data
Source: PLoS Comput Biol. 2024 Sep 23;20(9):e1012091. doi: 10.1371/journal.pcbi.1012091 (PMC11449291; doi:10.1371/journal.pcbi.1012091)
Supplement: Attachment — Submitted filename: Review_PLoS_CB.pdf [file pcbi.1012091.s017.pdf]

In this article, Dietler et al. perform a rigorous and systematic benchmark of statistical methods for identifying protein sectors in the presence of phylogeny. Protein sectors have been acknowledged as important signatures of functionally relevant sites that can be inferred from protein sequence statistics alone, but a systematic inspection of their information retrieval capacity in the presence of phylogeny and for varying degrees of selection was not presented before to my knowledge. In particular, the authors compare the standard definition of sectors to a more recent method with similar purpose (ICOD) and to conservation. They first analyse an in silico model of protein evolution under phylogenetic constraints, to get a full insight into the methods' performance in a fully controlled scenario; next they generalise their insights to a long list of real-data examples from protein families.

The work respects all standards of scientific and academic quality and deserves publication in a venue like PLoS CB. I have two major comments and a few minor remarks.

Major comments:

1. The methodological description of the comparison to DMS data is a bit too quick to be fully clear. It is fine to have it in the Methods only, but, for instance, why does one need to binarise these data? Is this binarised version that they later denote as 'DMS score'? I think some clarification here can help also understand how the symmetrised AUC is calculated: I don't see any mention of how the receiver operating characteristic (ROC) in first instance is constructed. Once one has the DMS scores and the eigenvector components, how does one calculate the true and false positives rates needed for the ROC? What is the threshold value to vary?

2. The prediction of mutational effects by the components of certain eigenvectors is a bit presented as something obvious, but I don't think in the literature it has been treated. If the authors know of any benchmark of the use of components of SCA eigenvectors for the prediction of mutational effects, then in the manuscript they should mention these studies to contextualise their analysis. On the other hand, if it has not been proposed and/or systematically benchmarked before, then it might be worth expanding the discussion to compare it to other methods that use single-site frequencies and correlations in protein sequence data; in particular I have in mind DCA, which has been tested for mutational effect predictions. Could the authors infer even a simple mean-field DCA from the protein MSAs (using same pseudo-counts, cutoffs etc), predict mutational effects by the energy difference and report this comparison to ICOD and conservation (i.e. the best performing methods)? I think this would be useful to the community to establish what strategy is most suitable for this task.

Minor remarks:

For the claim at the bottom of page 2, on sectors with positive and negative mutational effects, could the authors add the spectrum in the supplementary as supporting evidence?

Caption of Fig 2: why is k rescaled by D^2? This rescaling is also used in section S1.2, but it's not clear to me its role.

Page 10: With 15 MSAs per protein family obtained via 15 different cutoffs, it's not obvious that the best prediction is given by summing component by component the respective eigenvectors. Would it make sense also to calculate the different metrics (symmetrised AUC, recovery) for each cutoff and then look at the average and standard deviation of these metrics across the 15 cases? If each cutoff stands for a certain level of phylogenetic similarity, could it be interesting also to stratify these metrics by cutoff (i.e. phylogenetic similarity)?

Caption Figure 8: it is said that APC was applied only to ICOD and MI, but not SCA. Could the authors report as supplementary the performance without APC? Is it necessary for all families, or is it this correction to evaluate on a case-by-case basis?

Discussion page 12: ‘Conservation has been used a lot to identify functionally important sites, and thus, its good performance is not surprising’. I think some references should be added here, for instance papers like this: https://www.ncbi.nlm.nih.gov/pmc/articles/PMC4686965/

Page 14: can one provide a reference for the Jukes-Cantor distance?

Page 15: can one quickly mention what the reference sequence gauge consist of?

Eq 18 in Supplementary: do we need to approximate spins with continuous values to apply this?

Section S1.2: is it possible that there has been a switch of notation from L to N that has not been clarified?

Figure S1 caption: ‘rep.’ should be ‘resp.’

Figure S3: why isn’t there included the no phylogeny baseline?
