# Supplementary material for: Impact of phylogeny on the inference of functional sectors from protein sequence data
Source: PLoS Comput Biol. 2024 Sep 23;20(9):e1012091. doi: 10.1371/journal.pcbi.1012091 (PMC11449291; doi:10.1371/journal.pcbi.1012091)
Supplement: Attachment — Submitted filename: review.pdf [file pcbi.1012091.s018.pdf]

Review

- **Title**: Impact of phylogeny on the inference of functional sectors from protein sequence data
- **Journal**: PLOS Computational Biology

In this manuscript, the authors set out to:

- assess the capacity of different methods to identify functional sites in a sequence evolving under a non-linear additive trait;
- understand the effect of phylogeny and the robustness of different methods in this context;
- evaluate the performance of methods on a vast set of DMS experiments.

I think that this is a very good work that addresses important points of our understanding of the sequence-function relationship. However, in its current state, the manuscript has some issues that I state in my comments below. As a general comment, I think that the manuscript is too long and a bit too verbose at times. This affects the clarity of the presentation, as I was sometimes confused as what the authors consider important results and what the guiding idea of the work is. For what regards the scientific content, I have many questions that I wrote below, but this appears like sound and serious work to me. More precise comments can be found below.

Synthetic data

1. The type of trees used for simulations is not clearly defined. It seems from Figure 1 that the trees are perfectly balanced, but this is never stated explicitly.
   - If this is the case, it should be clearly stated.
   - Perfectly balanced tree are very particular because of their symmetry. I imagine that this structure has an influence over the eigenvalues of, say, the correlation matrix. Could the authors replicate some of their main results for other classes of trees (*e.g.* ladder, from a coalescent process, or inferred from data), and see if there is any qualitative change?

2. Three values of μ are used, but not a lot of context is given to help the reader understand why $\mu = 50$ and $\mu = 5$ correspond to respectively weak and strong phylogeny.
   - For these values of μ, how close are two typical sequences in the synthetic alignment? Two sequences corresponding to a cherry on the tree? How does this compare with the sequence alignments used in the part on DMS data? Maybe using μ/L as a control parameter would be clearer (where L is the sequence length)?

- For actual sequence data, parameter μ is unknown. Can the authors think of any observable or set of observables that could be computed on the sequences or on the inferred tree to give a sense of the strength of phylogenetic effects? For instance, something related to the pairwise hamming distance between sequences, or to the effective number of sequences? This may be a bit out of scope, but would help bridge the two parts between phylogenetic effects on synthetic data and prediction of mutational effects on real data.

3. As I understand it, the goal of ICOD and SCA is to identify a group of sites that are functionally important (sector). In the case where only one trait is selected, as in Equation 1, the authors show that this works very well (at least without phylogeny). However, in any real protein, I expect selection to be composed of possibly multiple traits and maybe specific pairwise interactions that stabilize the structure. How does the performance of ICOD generalize? Could the authors convince the reader, maybe using a model with more than one trait, that ICOD remains able to pick out functional sites even in the presence of phylogeny?

4. The authors refer many times to the eigenvalues/vectors on the "opposite" end of the spectrum: Λ_{min} for the ICOD matrix and λ_{max} for the correlation matrix. Yet, they also show that these generally perform worse. Do these eigenvectors contain different information than the main one, or just the same but worse? Unless there is a specific reason not to, I suggest sending most of the discussion on these to the supplement to make the flow of the main text smoother. This could help simplify Figure 4.

5. The part on the dependence of recovery on τ and κ is not very convincing. Many non-trivial effects are uncovered: ICOD reaches a minimum for intermediate values of τ, with a position that depends on μ; SCA has a plateau-like maximum, then drops for large τ; in Figure 6, results qualitatively differ for two values of τ, would a third value give us yet another different picture? In Figure S6, why is there a sharp drop in the performance of SCA for a specific value of τ? After reading this part two times, I still do not know what to think, and I have the feeling that the authors do not understand much more. I encourage the authors to clarify the presentation of this part.

6. The authors study the dependence of components of the top eigenvectors on the position G of the first mutation. I think this is a very interesting idea.
   - G is defined as a "generation", but I assume that it refers to the level of the tree at which the mutation happens? *i.e.* there are 2^{11-G} sequences concerned by the mutation?

- the y-axis scale on Figure 7 makes it hard to see what's happening for C and at low G: it could be that correlation still disentangles sector and non-sector sites.
- on Figure 5, for C and small μ, scores go up with G. The explanation given by the authors is that sites that mutate late have low variance and thus contribute slowly to the λ_{min} eigenvector. However, ICOD seems precisely designed to pick up low variance sites, as explained in the first paragraph of page 4. However, contributions of sites to the ICOD eigenvector only increase moderately with G. Could it be that the increase for C is due to conservation (since the diagonal is not removed)? If this is the case, maybe it should be stressed.

DMS data

7. This part is very interesting, and shows that ICOD and conservation both outperforms SCA at predicting functionally importantsites. However, the link with the part on simulated data is not so clear: do we observe these results because of the phylogenies corresponding to the alignments, or just because ICOD generally outperforms SCA?

8. For DMS data, authors use the AUC under a ROC as a metric, which as I understand it makes sense for a classifier (*i.e.* sector vs non-sector site). However, the DMS data consists of effects of single mutations, which can potentially be averaged to obtain site-specific effects. What do the authors use as the ground truth for this classifier? Does this have to do with the bar labelled "cutoff" in Figure S12? If so, how is this cutoff determined? And does it really make sense to try to identify a sector when the DMS looks like a gaussian like in the bottom row of Figure S12? I might misunderstand what the authors are doing but I did not find it well explained.

9. In Figure 8 it is said that the APC is used. One of the legends surrounding APC is that it in parts corrects for phylogeny. Did the authors try to use APC in their synthetic data to see what its effect is?

10. The authors state that ICOD and conservation use different information, which I would be inclined to believe. However, Table S1 shows that sector sites identified by both methods strongly overlap. Do the authors know why? A good argument for the fact that ICOD and conservation are different would be the point at $\tau = 0$ in Figure 5: conservation completely fails as expected, while ICOD is perfect.

Organization of the text

11. Maybe Figure 2 could be moved to the supplement. It is helpful to provide intuition as to how ICOD works, but also takes up space and reading time.

12. Figure 4 contains a lot of curve, but surprisingly does not show the performance of SCA. I would suggest removing the "no phylogeny" lines as the saturation of curves at high μ is quite clear, removing the Λ_{min} and λ_{max}, and adding the recovery of SCA. The link with the analysis of DMS data would then be clearer.

13. Many parameters influence results: target trait value τ, selection strength κ and number of mutations μ. I suggest that the values of parameters used for each figure be clearly stated in the caption (for instance as a tuple of value). Right now, this information is not practical for the reader to obtain.

14. Figures 5 is very difficult to parse due to the number of curves. An idea to make results more readable (but the authors may think of a better one) would be to *(i)* remove results on C, which at this point has been shown to be worse than ICOD, and *(ii)* have three subplot for the three values of μ, each with three curves (ICOD, SCA, conservation).

15. Figure 6: removing the markers might make it easier to distinguish the dashed and solid lines.

Minor comments, typos

- I could not understand how parameters D_i are chosen, although I may have missed the paragraph that explains it?
- Abstract, "Modeling showed that natural selection on an additive functional trait ...". Maybe this should be "*nonlinear* selection"?
- first page, "...which **have are** associated to a functional role..."
- page 4, "... the ICOD matrix **and its the** spectrum without phylogeny ..."
- page 13 "... we can also generate sequences **wit** phylogeny and no selection ..."
