# Supplementary material for: Impact of phylogeny on the inference of functional sectors from protein sequence data
Source: PLoS Comput Biol. 2024 Sep 23;20(9):e1012091. doi: 10.1371/journal.pcbi.1012091 (PMC11449291; doi:10.1371/journal.pcbi.1012091)
Supplement: Attachment — Submitted filename: Review_ Impact of phylogeny on sectors.pdf [file pcbi.1012091.s019.pdf]

Dear Editor,

We thank you for handling our manuscript and obtaining the valuable reports. We appreciate the useful and in-depth feedback by all three reviewers. We revised our manuscript according to their input, and we believe that it is substantially improved as a result, both in content and in readability.
In particular, we added an additional analysis that considers synthetic data with more diverse phylogenetic trees, as well as another one that relates the diversity of our synthetic data to that of natural data. We also streamlined our manuscript by moving the results on the impact of selection parameters in synthetic data to a Supplementary Appendix.
Below, we respond to each specific point raised by the reviewers. Our responses are in blue and preceded by ">>>". We also attach a version of our manuscript where our changes are highlighted in blue.
We hope that our revised manuscript will be found suitable for publication in PLOS Computational Biology.

Best regards,
Nicola Dietler, Alia Abbara, Subham Choudhury and Anne-Florence Bitbol

Reviewer 1:

In this article, Dietler et al. perform a rigorous and systematic benchmark of statistical methods for identifying protein sectors in the presence of phylogeny. Protein sectors have been acknowledged as important signatures of functionally relevant sites that can be inferred from protein sequence statistics alone, but a systematic inspection of their information retrieval capacity in the presence of phylogeny and for varying degrees of selection was not presented before to my knowledge. In particular, the authors compare the standard definition of sectors to a more recent method with similar purpose (ICOD) and to conservation. They first analyse an in silico model of protein evolution under phylogenetic constraints, to get a full insight into the methods' performance in a fully controlled scenario; next they generalise their insights to a long list of real-data examples from protein families.

The work respects all standards of scientific and academic quality and deserves publication in a venue like PLoS CB. I have two major comments and a few minor remarks.

>>> We thank the reviewer for the positive assessment and for the very relevant suggestions which allowed us to improve our manuscript.

Major comments:

1. The methodological description of the comparison to DMS data is a bit too quick to be fully clear. It is fine to have it in the Methods only, but, for instance, why does one need to

binarise these data? Is this binarised version that they later denote as ‘DMS score’? I think some clarification here can help also understand how the symmetrised AUC is calculated: I don’t see any mention of how the receiver operating characteristic (ROC) in first instance is constructed. Once one has the DMS scores and the eigenvector components, how does one calculate the true and false positives rates needed for the ROC? What is the threshold value to vary?

>>> The binarization of the data allows defining sector and non-sector sites via a threshold. We now added the following sentence in the paragraph called "Inferring mutational effects" of Methods to motivate this point: "While in principle we can infer mutational effect values from eigenvectors at least with ICOD [31], the measured fitness can involve additional nonlinearities not captured by our simple model."

Besides, we apologize for being slightly cryptic about the details of our analysis of natural data. We now added some specifics in the paragraph titled "Comparing to experiments" of Methods.
Regarding how we binarize the data, we added: "To define the set of sites with large mutational effects (which corresponds to the sector), we use a threshold on the DMS measurements to binarise them, leading sector and non-sector sites. The threshold is set as follows (...)"
Regarding how we calculate the AUC, we added: "Once the sector and non-sector sites are defined, the performance of various models at identifying sector sites is quantified by the symmetrized AUC. See the paragraph on “Performance evaluation” below for details."
And in the paragraph on “Performance evaluation” of Methods, we added "To calculate the AUC, we vary the threshold on the numbers of sites predicted (which are the top eigenvector components). Specifically, computing the true positive and false positive rates for each threshold value allows constructing the receiver operating characteristic curve and computing the AUC."

2. The prediction of mutational effects by the components of certain eigenvectors is a bit presented as something obvious, but I don’t think in the literature it has been treated. If the authors know of any benchmark of the use of components of SCA eigenvectors for the prediction of mutational effects, then in the manuscript they should mention these studies to contextualise their analysis. On the other hand, if it has not been proposed and/or systematically benchmarked before, then it might be worth expanding the discussion to compare it to other methods that use single-site frequencies and correlations in protein sequence data; in particular I have in mind DCA, which has been tested for mutational effect predictions. Could the authors infer even a simple mean-field DCA from the protein MSAs (using same pseudo-counts, cutoffs etc), predict mutational effects by the energy difference and report this comparison to ICOD and conservation (i.e. the best performing methods)? I think this would be useful to the community to establish what strategy is most suitable for this task.

>>> As pointed out in our response above, in our analysis of natural data, the binarization of the data allows defining sector and non-sector sites via a threshold. The sector and non-sector sites are then predicted using eigenvectors of the SCA or covariance or ICOD matrix. This use of eigenvectors is more in line with the traditional uses of SCA. We now added the following sentence in the paragraph called "Inferring mutational effects" of

Methods to clarify this point: "Note that for natural data, we do not aim to infer mutational effect values but only their rank, in line with the usual practice of SCA [27, 29]." Nevertheless, it was shown in Ref. [31] that the components of the top eigenvector of the ICOD matrix reveals mutational effects within a simple model. But nonlinearities mean that it is difficult to fully translate this to natural data.

We thank the reviewer for raising the interesting point of a comparison to DCA energy for sector prediction. Since the DMS data we considered was previously used to infer mutational effects via DCA in Ref. [46], we briefly compared our results to theirs. In the figure below, we show a comparison of the performance of ICOD and scores using the statistical energy of a DCA inferred model. Note that the MSAs used in Riesselmann et al. [46] are different from the ones used in our article, both in terms of sequences and in terms of sites (length of sequences), which makes the comparison not entirely fair. We find that the average performance of ICOD is 0.42 and the DCA inferred energies performance is 0.47. Thus, the performance appears to be slightly better using inferred energies. Nevertheless, eigenvector-based methods such as SCA have the advantage that they can distinguish different aspects of function and functional constraints via different eigenvectors.

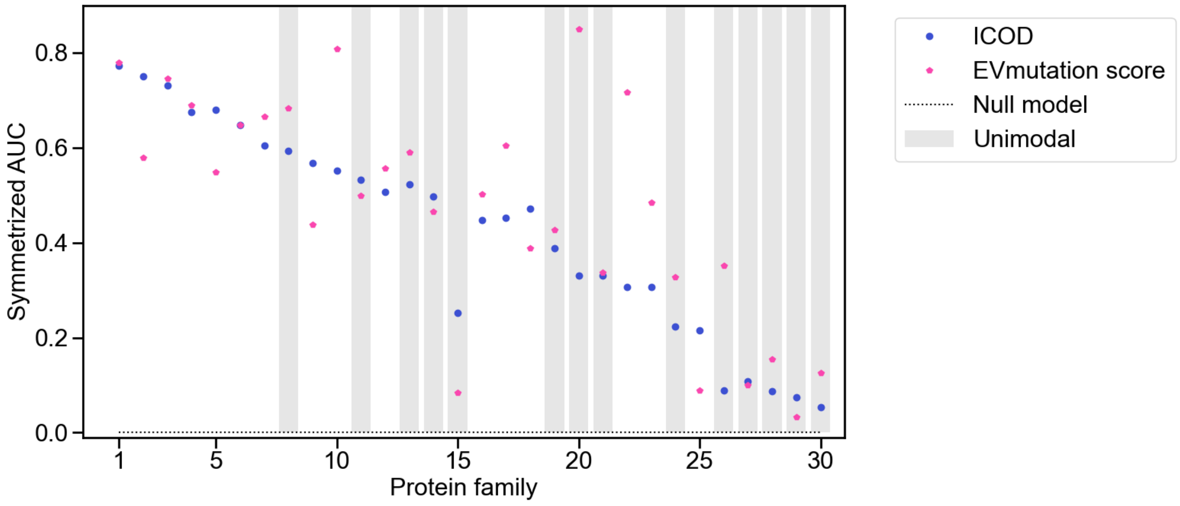

We added the following sentences in the Discussion: "Methods able to predict mutational effects can be used to determine sites with top mutational effects. We compared using the ICOD eigenvector and using the Potts model energy for this as in [46], and found that the latter had slightly better average symmetrized AUC. Note however that the results of [46] were obtained using different MSAs, which makes the comparison imperfect. We stress that eigenvector-based methods, such as SCA and ICOD, have the potential to disentangle different aspects of function via different eigenvectors [27,30,31], which is not the case of Potts energy-based methods. Studying the impact of phylogeny on cases with simultaneous selection on multiple traits, as in Ref. [31], would be interesting."

Minor remarks:

For the claim at the bottom of page 2, on sectors with positive and negative mutational effects, could the authors add the spectrum in the supplementary as supporting evidence?

>>> We now added Figure S2 to address this point. It is very similar to Figure 2.

Caption of Fig 2: why is k rescaled by D^2? This rescaling is also used in section S1.2, but it's not clear to me its role.

>>> Our goal in this element of the caption of Figure 2 is simply to set a scale for kappa that makes it comparable with the other scales involved in the selection model. Meanwhile, in section S1.2, rescaled parameters are used to match the problem solved in "High-temperature expansions and message passing algorithms" by Maillard et al. In both cases, these scalings are employed for convenience.

Page 10: With 15 MSAs per protein family obtained via 15 different cutoffs, it's not obvious that the best prediction is given by summing component by component the respective eigenvectors. Would it make sense also to calculate the different metrics (symmetrised AUC, recovery) for each cutoff and then look at the average and standard deviation of these metrics across the 15 cases? If each cutoff stands for a certain level of phylogenetic similarity, could it be interesting also to stratify these metrics by cutoff (i.e. phylogenetic similarity)?

>>> We thank the reviewer for raising this interesting point. We agree that summing component by component the various eigenvectors is not the only way to aggregate results over phylogenetic cutoffs. We performed the analysis suggested by the reviewer and added its results in Figure S8 of the revised manuscript. Overall, the results are consistent but slightly less good. We added the following sentences to the main text: "Note that there are other ways of aggregating scores across phylogenetic cutoffs. Averaging performance metrics across cutoffs gives results that are overall consistent with those obtained by summing eigenvector components, but slightly less good, see Fig S8. "

In the figure below, we further show a scatter plot of the scores using the sum of each eigenvector from each cutoff versus the average score over cutoffs. The Pearson correlation coefficients are shown in the legend of the figure. These results confirm that observations are similar using both ways of aggregating scores over phylogenetic cutoffs.

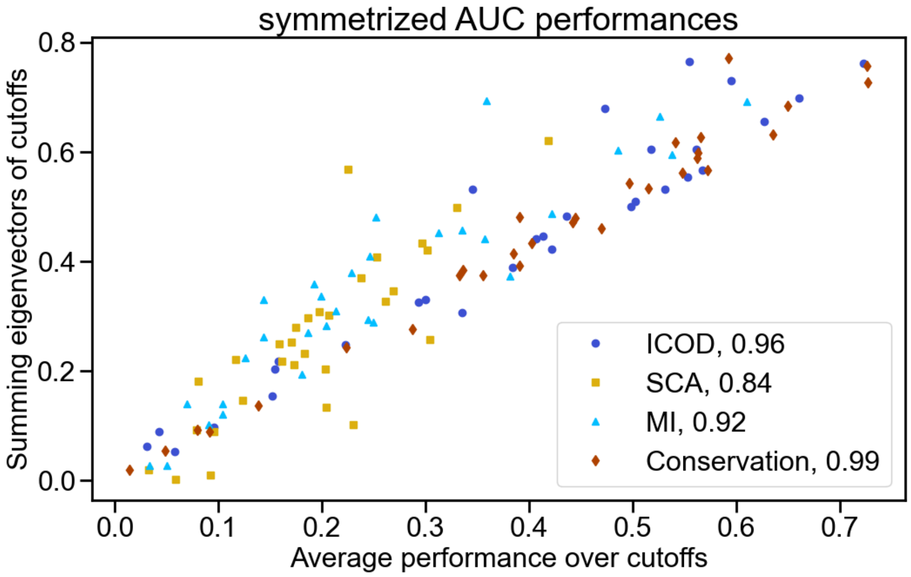

Caption Figure 8: it is said that APC was applied only to ICOD and MI, but not SCA. Could the authors report as supplementary the performance without APC? Is it necessary for all families, or is it this correction to evaluate on a case-by-case basis?

>>> In the figure below, we report the performance of all methods without APC. Compared to Figure 6 of our manuscript, we observe that the performance is deteriorated for ICOD and MI. The average performance of ICOD and MI with APC is 0.43 and 0.35 respectively, and without APC it becomes 0.36 and 0.27 respectively. For most protein families considered, the performance of ICOD and MI is enhanced when using APC. Conversely, the performance of SCA is deteriorated by APC, indeed APC leads to an average performance of 0.13 for SCA.

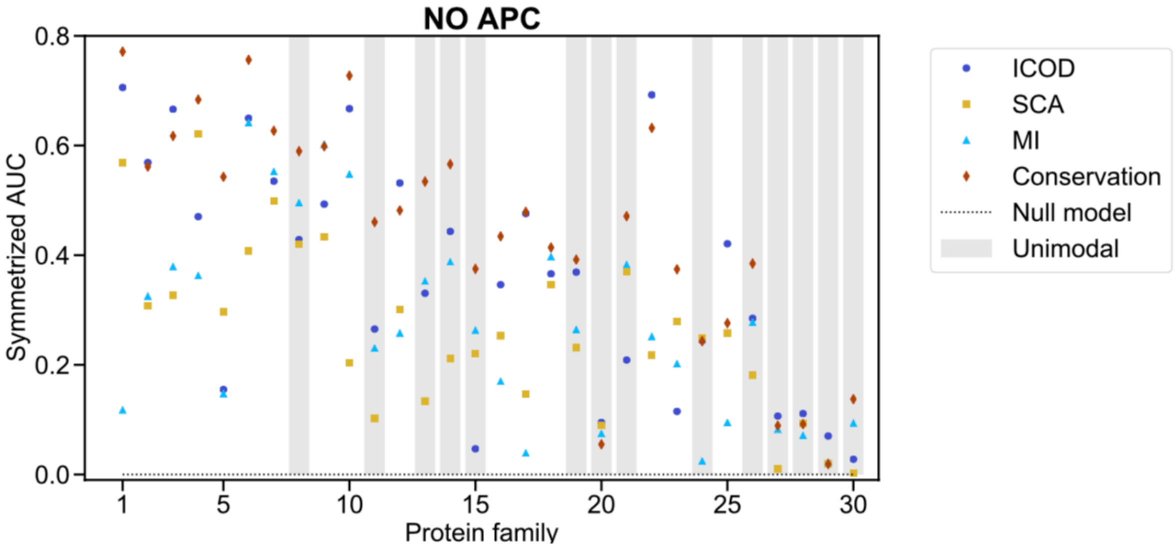

Because we already have a large number of supplementary figures, we chose not to add this one, and to rather add an extra specific in the following sentence of the legend of Figure 6: "For ICOD and MI, the average product correction (APC) [4] is applied to the

matrix of interest (it was found to improve the average performance for most families for these methods, but not for SCA)."

Finally, the reviewer is correct that APC could potentially be applied or not to a given family on a case-by-case basis. However, it would be difficult to do so for predictive purposes, which is why we chose to apply it either for all families or for none with each method considered.

Discussion page 12: ‘Conservation has been used a lot to identify functionally important sites, and thus, its good performance is not surprising’. I think some references should be added here, for instance papers like this:
https://www.ncbi.nlm.nih.gov/pmc/articles/PMC4686965/

>>> We added the relevant reference suggested by the reviewer.

Page 14: can one provide a reference for the Jukes-Cantor distance?

>>> We added a reference as suggested by the reviewer.

Page 15: can one quickly mention what the reference sequence gauge consist of?

>>> We now specify: "The reference-sequence gauge [31] is used, meaning that the reference sequence of the dataset (i.e., the wild-type sequence for deep mutational scan data) is chosen as our baseline."

Eq 18 in Supplementary: do we need to approximate spins with continuous values to apply this?

>>> While the auxiliary field h is continuous, the spins can remain discrete. We added a reference about the Hubbard-Stratonovitch transform for interested readers.

Section S1.2: is it possible that there has been a switch of notation from L to N that has not been clarified?

>>> We thank the reviewer for noticing this unfortunate inconsistency in our notations. We corrected it, and now the sequence length is called L throughout.

Figure S1 caption: ‘rep.’ should be ‘resp.’

>>> We thank the reviewer for noticing this typo. We corrected it.

Figure S3: why isn’t there included the no phylogeny baseline?

>>> We had removed it just for reasons of legibility. However, we have now modified and improved the presentation of these results, and this allowed us to include the no phylogeny baseline, as suggested by the reviewer. The corresponding figures are Figure 4 and Figure S3 in the revised manuscript.

Reviewer 2:

This paper explores the impact of phylogeny on the inference of protein sectors using different methods: ICOD, covariance, conservation, and SCA. For this, the authors conducted an exhaustive study on synthetic data generated by a minimal model of sequences with functional sectors and a phylogenetic tree. The results enabled the dissection of phylogeny's role in selection signals and the establishment of a method hierarchy based on phylogeny robustness for sector identification and mutational effect inference. The insights derived from synthetic data analysis helped to interpret important site identification in natural protein families using the methods under study.

The authors address the problem rigorously, and their main result is that predictions with ICOD and conservation methods are more accurate due to their robustness to phylogeny. Although their work is sound and convincing, a few points require further clarification.

>>> We thank the reviewer for the positive assessment and for the very relevant suggestions which allowed us to improve our manuscript.

1- Conclusions derived from Figure 3 on the impact of phylogeny in matrices spectra are based on the observation or not of contrast of the outlier eigenvalue with the rest of the spectrum. However, this is done for a single simulation of the evolutionary process; how robust is this to fluctuations due to phylogenetic sampling? For instance, it is not clear why, if phylogeny impacts large eigenvalues of the covariance matrix, the contrast of its smaller eigenvalue with the rest of the spectrum decreases for strong phylogeny. Is this just sampling noise?

>>> These observations are reproducible and robust to sampling noise. We now added Figure S2 which shows another single realization in a slightly different case, where some mutational effects are negative. It is very similar to figure 2. We further performed 100 replicates, computed the spectra for each of them and then plotted the average of the ordered eigenvalues over all these replicates. The figure below is again very similar to figure 2, showing that the conclusions are robust.

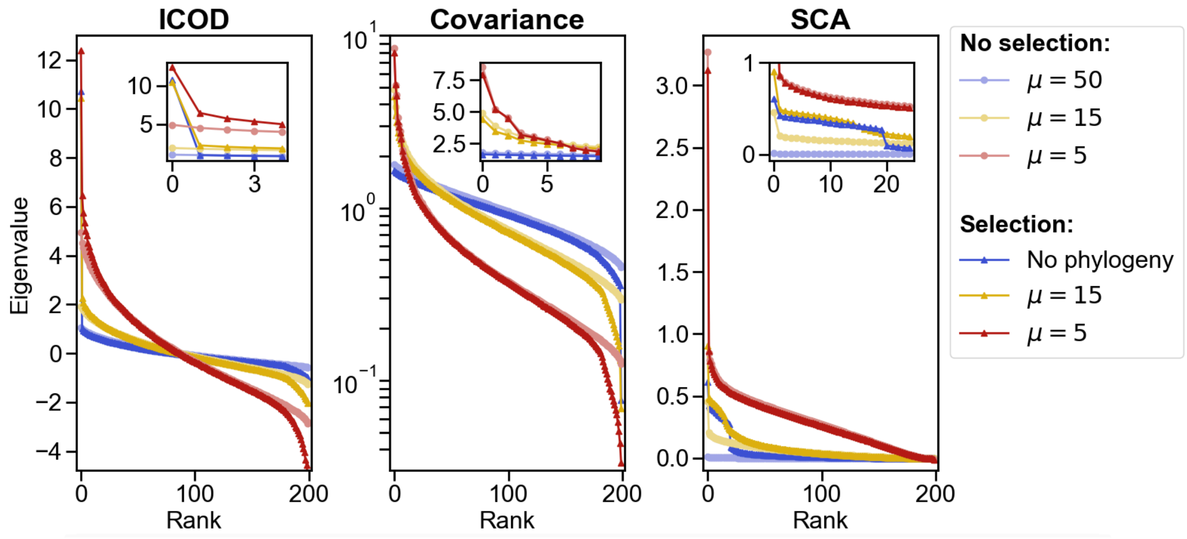

Regarding the reviewer's specific remark about small vs. large eigenvalues, phylogeny

affects the covariance matrix globally, but it is true that there are particularly large (and known) signatures in large eigenvalues.

2- The description of Figure 6 does not explain why the recovery decreases for the covariance matrix when selection strength increases for \tau*=140 without the presence of phylogeny. I guess this is because increased conservation and covariance are susceptible to it in contrast with the ICOD method; however, it would be recommended to explain this in the text for completeness.

>>> We agree with the reviewer on the interpretation of the behavior observed in this figure, which is now Figure VII in section 2.2 of the Supplementary Appendix. To specify this, we added the following sentence "Even in the absence of phylogeny, recovery by covariance decreases as selection strength increases for tau*=140. This can also be attributed to increasing conservation, to which ICOD is more robust. "

3- In the discussion section of the article, it is suggested that the superiority of the conservation method over SCA in the analysis of natural protein families is a result of its robustness to phylogenetic correlations. However, for synthetic data, it was found that this is only possible under strong phylogenetic regimes, which is unlikely in the case of homologous protein families and, in particular, for all the families studied. Could the authors elaborate on this hypothesis?

>>> We thank the reviewer for raising this important point.

In synthetic data, we observe a complex interplay between phylogeny (tuned by mu) and selection (tuned by tau* and kappa). For instance, in Figure II in section 2.1 of the Supplementary Appendix, we observe that for small tau*, ICOD always outperforms both conservation and SCA - both with strong phylogeny and without phylogeny.

More generally, the question of how the robustness of ICOD to phylogeny observed in synthetic data translates to natural data is definitely important. It is not straightforward to make an exact mapping between the regimes and parameters of our minimal model and natural data, because of the various simplifications involved in the model. Nevertheless, we performed two additional analyses to clarify this link:

- First, we added Table S2 which gives some numbers on the diversity and depths of the natural MSAs we considered, specifically in the form of mean pairwise Hamming distances, depths and effective depths. We also computed the mean pairwise Hamming distances in our synthetic datasets, and they are comparable. We thus added the following sentences in the section titled "Impact of phylogenetic correlations" of Results: "The mean pairwise Hamming distances in these synthetic datasets are 0.47 for mu=50 and 0.30 for mu=5. They fall in the range observed in natural data, see Tables S1 and S2."
- Second, we added Figure S10, which shows how the relative difference of performance observed between ICOD and SCA in natural MSAs depends on the mean Hamming distance in these MSAs. We observed that ICOD most outperformed SCA for the least diverse MSAs considered, which is in line with our hypothesis that the robustness of ICOD to phylogeny is an important ingredient of its performance on natural data.

We thus added the following paragraph in the section titled "Identifying functionally important sites in natural protein families" of Results: "The observation that ICOD and conservation tend to outperform SCA is consistent with our results obtained in synthetic data. To assess more directly the link between these results and phylogeny in natural data, Fig S10 shows the relative difference of ICOD and SCA performances versus MSA diversity, characterized by the mean pairwise Hamming distance between sequences in the MSA (see also Table S2). We find that for all 7 families with mean pairwise Hamming distance smaller than 0.55, ICOD significantly outperforms SCA, while results are more mixed for more diverse families. These 7 least diverse families are precisely those for which we expect the effect of phylogeny to be strongest. This result suggests that phylogeny does explain part of the success of ICOD on these natural datasets."

We believe that these additions strengthen our manuscript.

4- A primary result of this work is the demonstration that the ICOD method is consistently more robust than other methods in the presence of correlations of phylogenetic origin. However, from the manuscript, it is understood that pseudocounts regularization is used only for the ICOD method during covariance matrix calculation, while for other methods, it is not. Pseudocount is a regularization scheme developed to correct for non-invertible matrices due to finite sampling, an effect to which phylogenetic correlations also contribute by reducing the effective number of sequences. At the same time, pseudocount has been shown to improve the predictions of other methods that do not necessarily require the inversion of the covariance matrix but use the information it contains. If the above is correct, is it possible that pseudocount is a way to reduce the bias of phylogenetic correlations, and therein lies an advantage over the other methods? Could the authors verify that this is not what makes the difference?

>>> As rightly pointed out by the reviewer, our use of a small pseudocount (1e-5 in practice) in ICOD is motivated by the need to invert the covariance matrix in this method. In the figure below, we reproduce figure 4 of our revised manuscript, but including the same pseudocount values in all methods, i.e. also for covariance, SCA and conservation. As can be observed, the figure is almost unchanged, confirming that the pseudocount does not play an important role here.

We added the following sentences in the section titled "Impact of phylogeny on mutational effect recovery" of Results, where we also mentioned the quantitative impact of adding a pseudocount in other methods than ICOD: "In Fig 4 and throughout, we employ a small pseudocount for ICOD but not for other approaches (see Methods). This is motivated by the need to invert the covariance matrix in ICOD [31]. For covariance, the difference in the recovery between results with and without pseudocount (taking the same pseudocount value as for ICOD) is lower than 1.2% for all values of mu. For SCA, it is lower than 0.3%, and for conservation it is lower than 3x10^(-4)%. Thus, the pseudocount does not affect the conclusions."

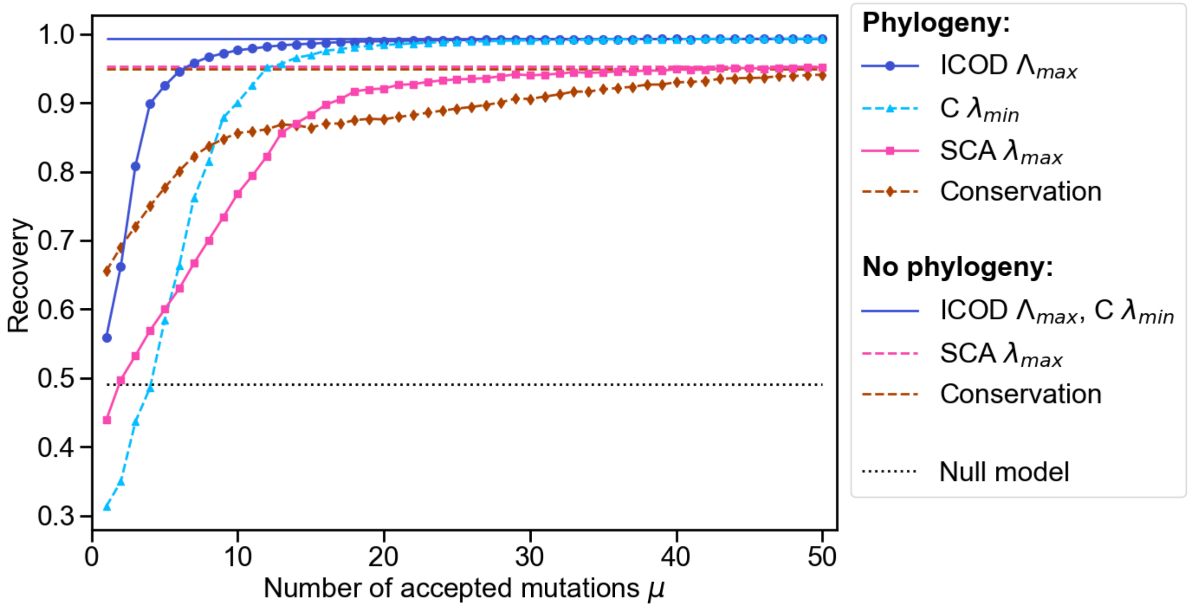

5- During the synthetic data study, a binary, symmetric, and homogeneous tree was considered. However, the spectrum of eigenvalues of the covariance matrix is known to be modified for more realistic phylogenies, although a power law tail of large eigenvalues is maintained. Could the authors discuss the effects of this simplification on the paper's conclusions and how it would impact a typical tree of natural data, which is inhomogeneous and has multiple branching events?

>>> We thank the reviewer for asking this interesting question.
We performed a new analysis to address it, whose results are presented in the new Figures S5 and S6, and described in a new dedicated paragraph titled "Generalization to other phylogenetic trees" within our section titled "Impact of phylogeny on mutational effect recovery" of Results. Briefly, we considered a family of phylogenetic trees known as Beta-coalescent trees, which can reflect either neutral evolution or evolution under selection depending on a parameter. We found that our main results are robust to considering diverse phylogenetic trees. We discuss further details of the analysis in the dedicated paragraph.
We believe that this addition strengthens our manuscript.

Reviewer 3:

In this manuscript, the authors set out to:

- assess the capacity of different methods to identify functional sites in a sequence evolving under a non-linear additive trait;
- understand the effect of phylogeny and the robustness of different methods in this context;
- evaluate the performance of methods on a vast set of DMS experiments.

  I think that this is a very good work that addresses important points of our understanding of the sequence-function relationship. However, in its current state, the manuscript has some issues that I state in my comments below. As a general comment, I think that the manuscript is too long and a bit too verbose at times. This affects the clarity of the presentation, as I was sometimes confused as what the authors consider important results and what the guiding idea of the work is. For what regards the scientific content, I have many questions that I wrote below, but this appears like sound and serious work to me. More precise comments can be found below.

>>> We thank the reviewer for the positive assessment and for the very relevant suggestions which allowed us to improve our manuscript.

The main topic of this manuscript regards the impact of phylogeny on the inference of functional sectors. In synthetic data, we observed a complex interplay between phylogeny (tuned by mu) and selection (tuned by tau* and kappa), which drove us to analyze it. However, we agree that it is not central to our manuscript and may distract the reader from the main point. Thus, in our revised version, we moved this analysis to our Supplementary Appendix. We believe that this streamlines our manuscript.

Synthetic data

1. The type of trees used for simulations is not clearly defined. It seems from Figure 1 that the trees are perfectly balanced, but this is never stated explicitly.

   - If this is the case, it should be clearly stated.
   - Perfectly balanced tree are very particular because of their symmetry. I imagine that this structure has an influence over the eigenvalues of, say, the correlation matrix. Could the authors replicate some of their main results for other classes of trees (e.g. ladder, from a coalescent process, or inferred from data), and see if there is any qualitative change?

>>> As understood by the reviewer, we considered perfect binary trees. We now added "perfect" throughout to make this clear. We apologize for being slightly imprecise in our wording.

We thank the reviewer for asking the interesting question of the extension to more general phylogenies.
We performed a new analysis to address it, whose results are presented in the new Figures S5 and S6, and described in a new dedicated paragraph titled "Generalization to other phylogenetic trees" within our section titled "Impact of phylogeny on mutational effect recovery" of Results. Briefly, we considered a family of phylogenetic trees known as Beta-coalescent trees, which can reflect either neutral evolution or evolution under selection depending on a parameter. We found that our main results are robust to considering diverse phylogenetic trees. We discuss further details of the analysis in the dedicated paragraph.
We believe that this addition strengthens our manuscript.

2. Three values of μ are used, but not a lot of context is given to help the reader understand why μ = 50 and μ = 5 correspond to respectively weak and strong phylogeny.

- For these values of μ, how close are two typical sequences in the synthetic alignment? Two sequences corresponding to a cherry on the tree? How does this compare with the sequence alignments used in the part on DMS data? Maybe using μ/L as a control parameter would be clearer (where L is the sequence length)?

- For actual sequence data, parameter μ is unknown. Can the authors think of any observable or set of observables that could be computed on the sequences or on the inferred tree to give a sense of the strength of phylogenetic effects? For instance, something related to the pairwise hamming distance between sequences, or to the effective number of sequences? This may be a bit out of scope, but would help bridge the two parts between phylogenetic effects on synthetic data and prediction of mutational effects on real data.

>>> We thank the reviewer for raising these important points.
We added specifics at the beginning of Results on distances within our trees: "A fixed number mu of accepted mutations are performed on each branch. As a result, two closest (resp. farthest) sequences on the leaves of a tree differ at most by 2mu mutations (resp. 2mu n mutations, where n is the number of generations, i.e. of branching events, in the tree). Note that these numbers are reduced if multiple substitutions occur at the same site."
We further computed the mean pairwise Hamming distances in our synthetic datasets, and added the following sentences in the section titled "Impact of phylogenetic correlations" of Results: "The mean pairwise Hamming distances in these synthetic datasets are 0.47 for mu=50 and 0.30 for mu=5."
Besides, we added Table S2 which gives some numbers on the diversity and depths of the natural MSAs we considered, specifically in the form of mean pairwise Hamming distances, depths and effective depths. We observe that the Hamming distances in our

synthetic alignments (0.47 for mu=50 and 0.30 for mu=5) are in the range of these natural values.

3. As I understand it, the goal of ICOD and SCA is to identify a group of sites that are functionally important (sector). In the case where only one trait is selected, as in Equation 1, the authors show that this works very well (at least without phylogeny). However, in any real protein, I expect selection to be composed of possibly multiple traits and maybe specific pairwise interactions that stabilize the structure. How does the performance of ICOD generalize? Could the authors convince the reader, maybe using a model with more than one trait, that ICOD remains able to pick out functional sites even in the presence of phylogeny?

   >>> We agree with the reviewer that the generalization of ICOD and SCA to more than one functional trait under selection is highly interesting. This was investigated by one of us in Wang et al. [31] without phylogeny. We could in principle analyze the impact of phylogeny on these more complex cases. However we already have the feeling (which seems shared by the reviewer) that our manuscript is long, and fear that adding other parameters would make it too long. Thus, we preferred to leave this for future work, and we added a mention of this very interesting point in the Discussion: "We stress that eigenvector-based methods, such as SCA and ICOD, have the potential to disentangle different aspects of function via different eigenvectors [27,30,31], which is not the case of Potts energy-based methods. Studying the impact of phylogeny on cases with simultaneous selection on multiple traits, as in Ref. [31], would be interesting. "

4. The authors refer many times to the eigenvalues/vectors on the "opposite" end of the spectrum: Λmin for the ICOD matrix and λmax for the correlation matrix. Yet, they also show that these generally perform worse. Do these eigenvectors contain different information than the main one, or just the same but worse? Unless there is a specific reason not to, I suggest sending most of the discussion on these to the supplement to make the flow of the main text smoother. This could help simplify Figure 4.

   >>> We thank the reviewer for this suggestion, which allowed us to clarify our figures. We now include only the key end of the spectrum in our revised version of Figure 4, and the opposite end of the spectrum in Figure S3. We believe it also simplified our discussion of the corresponding results.
   Regarding whether the eigenvectors at the opposite end of the spectrum contain different information or just the same but worse, it is difficult to fully answer. However, our analysis of the block diagonal approximation of the ICOD matrix in the section titled "Impact of selection and phylogeny on ICOD, covariance and SCA spectra" of Results gives partial insight, in the sense that the two ends of the spectrum tend to be informative about the sector.

5. The part on the dependence of recovery on τ and κ is not very convincing. Many non-trivial effects are uncovered: ICOD reaches a minimum for intermediate values of τ, with a position that depends on μ; SCA has a plateau-like maximum, then drops for large τ; in Figure 6, results qualitatively differ for two values of τ, would a third value give us yet another different picture? In Figure S6, why is there a sharp drop in the performance of SCA for a specific value of τ? After reading this part two times, I still do not know what to think, and I have the feeling

that the authors do not understand much more. I encourage the authors to clarify the presentation of this part.

>>> As mentioned above, the main topic of this manuscript regards the impact of phylogeny on the inference of functional sectors. In synthetic data, we observed a complex interplay between phylogeny (tuned by mu) and selection (tuned by tau* and kappa), which drove us to analyze it. However, we agree that it is not central to our manuscript and may distract the reader from the main point. Thus, in our revised version, we moved this analysis to our Supplementary Appendix. We believe that this streamlines our manuscript.

We also believe that this reorganization helps to clarify this complex discussion, partly because all related figures are now presented at the same place. In particular, we tried to explain the surprising behaviors around the specific value of tau* and showed Figure S9 (now Figure VI in section 2 of the Supplementary Appendix) for this purpose.

6. The authors study the dependence of components of the top eigenvectors on the position G of the first mutation. I think this is a very interesting idea.

- G is defined as a “generation”, but I assume that it refers to the level of the tree at which the mutation happens? i.e. there are 2^{11-G} sequences concerned by the mutation?

>>> The reviewer is correct. We clarified this point by writing in the caption of Figure 2: "We evolve it on a perfect binary tree over a fixed number n of "generations" (i.e., tree levels, corresponding to branching events), here n=2 generations." We also added that generations mean branching events at other points in the text.

- the y-axis scale on Figure 7 makes it hard to see what’s happening for C and at low G: it could be that correlation still disentangles sector and non- sector sites.

>>> We added an inset to this figure (now figure 5) for better visualization. As can be seen in the inset, covariance C does not disentangle sector and non-sector sites when mu=5.

- on Figure 5, for C and small μ, scores go up with G. The explanation given by the authors is that sites that mutate late have low variance and thus contribute slowly to the λmin eigenvector. However, ICOD seems precisely designed to pick up low variance sites, as explained in the first paragraph of page 4. However, contributions of sites to the ICOD eigenvector only increase moderately with G. Could it be that the increase for C is due to conservation (since the diagonal is not removed)? If this is the case, maybe it should be stressed.

>>> We agree with the reviewer’s interpretation and added a sentence to make this clearer: "Recall that while the eigenvector associated to the largest ICOD eigenvalue also focuses on small variances, the diagonal of the ICOD matrix is set to 0 in order to remove conservation effects. Consistently, the contributions of sites to the ICOD eigenvector only increase moderately with G."

DMS data

7. This part is very interesting, and shows that ICOD and conservation both outperforms SCA at predicting functionally important sites. However, the link with the part on simulated data is not so clear: do we observe these results because of the phylogenies corresponding to the alignments, or just because ICOD generally outperforms SCA?

   >>> We thank the reviewer for asking this important question. While it is difficult to completely settle it, we performed an additional analysis that partly addresses it. Specifically, we added Figure S10, which shows how the relative difference of performance observed between ICOD and SCA in natural MSAs depends on the mean Hamming distance in these MSAs. We observed that ICOD most outperformed SCA for the least diverse MSAs considered, which is in line with our hypothesis that the robustness of ICOD to phylogeny is an important ingredient of its performance on natural data.

   We thus added the following paragraph in the section titled "Identifying functionally important sites in natural protein families" of Results: "The observation that ICOD and conservation tend to outperform SCA is consistent with our results obtained in synthetic data. To assess more directly the link between these results and phylogeny in natural data, Fig S10 shows the relative difference of ICOD and SCA performances versus MSA diversity, characterized by the mean pairwise Hamming distance between sequences in the MSA (see also Table S2). We find that for all 7 families with mean pairwise Hamming distance smaller than 0.55, ICOD significantly outperforms SCA, while results are more mixed for more diverse families. These 7 least diverse families are precisely those for which we expect the effect of phylogeny to be strongest. This result suggests that phylogeny does explain part of the success of ICOD on these natural datasets."

   We believe that this addition strengthens our manuscript.

8. For DMS data, authors use the AUC under a ROC as a metric, which as I understand it makes sense for a classifier (i.e. sector vs non-sector site). However, the DMS data consists of effects of single mutations, which can potentially be averaged to obtain site-specific effects. What do the authors use as the ground truth for this classifier? Does this have to do with the bar labelled “cutoff” in Figure S12? If so, how is this cutoff determined? And does it really make sense to try to identify a sector when the DMS looks like a gaussian like in the bottom row of Figure S12? I might misunderstand what the authors are doing but I did not find it well explained.

   >>> We apologize for being slightly cryptic about the details of our analysis of natural data.

   First, we binarized DMS data to define sector and non-sector sites via a threshold. We now added the following sentence in the paragraph called "Inferring mutational effects" of Methods to motivate this point: "While in principle we can infer mutational effect values from eigenvectors at least with ICOD [31], the measured fitness can involve additional nonlinearities not captured by our simple model."

   Besides, we now added some specifics in the paragraph titled "Comparing to experiments" of Methods.

Regarding how we binarize the data, we added: "To define the set of sites with large mutational effects (which corresponds to the sector), we use a threshold on the DMS measurements to binarise them, leading sector and non-sector sites. The threshold is set as follows (...)"
Regarding how we calculate the AUC, we added: "Once the sector and non-sector sites are defined, the performance of various models at identifying sector sites is quantified by the symmetrized AUC. See the paragraph on “Performance evaluation” below for details."
And in the paragraph on “Performance evaluation” of Methods, we added "To calculate the AUC, we vary the threshold on the numbers of sites predicted (which are the top eigenvector components). Specifically, computing the true positive and false positive rates for each threshold value allows constructing the receiver operating characteristic curve and computing the AUC."

9. In Figure 8 it is said that the APC is used. One of the legends surrounding APC is that it in parts corrects for phylogeny. Did the authors try to use APC in their synthetic data to see what its effect is?

   >>> Applying APC on our synthetic data would actually remove most of the signal coming from the vector **D** of mutational effects, given the approximate form of the ICOD matrix as an outer product of **D** with itself, which we detail in section S1 of the Supplementary Appendix. We tried this and obtained poor results as expected.
   Nevertheless, we empirically found APC to be generally useful for natural data when employing ICOD and MI, and thus we used it there - see our response to reviewer 1's question starting with "Caption Figure 8" for more details.

10. The authors state that ICOD and conservation use different information, which I would be inclined to believe. However, Table S1 shows that sector sites identified by both methods strongly overlap. Do the authors know why? A good argument for the fact that ICOD and conservation are different would be the point at $\tau = 0$ in Figure 5: conservation completely fails as expected, while ICOD is perfect.

    >>> We agree that there is substantial overlap, and we believe it is because important sites tend to be both conserved and correlated. We added a remark about this in the main text: "There is often substantial overlap between sites found by ICOD and by conservation, which suggests that in practice many important sites are both somewhat conserved and somewhat correlated with others."
    We thank the reviewer for the cogent remark about the point at tau*=0 in Figure 5 (now Figure II in the Supplementary Appendix). We added an explicit mention of it in the section titled "Identifying functionally important sites in natural protein families" of Results: "Accordingly, with synthetic data, we observe in Fig II in section S2 of the Supplementary Appendix that ICOD reaches very good performance for a favored trait value tau*=0, such that conservation is uninformative. "

Organization of the text

11. Maybe Figure 2 could be moved to the supplement. It is helpful to provide intuition as to how ICOD works, but also takes up space and reading time.

>>> We considered this suggestion, but we preferred to move the whole sections on the interplay between phylogeny and selection (i.e. the discussions of the impact of tau* and kappa) to the Supplementary Appendix. Indeed we believe that the intuition provided by Figure 2 is valuable to understand what follows, while the discussion on the impact of tau* and kappa adds another layer of complexity.

12. Figure 4 contains a lot of curve, but surprisingly does not show the performance of SCA. I would suggest removing the “no phylogeny” lines as the saturation of curves at high μ is quite clear, removing the Λmin and λmax, and adding the recovery of SCA. The link with the analysis of DMS data would then be clearer.

>>> As mentioned above, we modified both Figure 4 and Figure S3. In particular we now include SCA in Figure 4, as recommended by the reviewer. We agree that it is important and helps with the analysis. We thank the reviewer for the suggestion.

13. Many parameters influence results: target trait value τ, selection strength κ and number of mutations μ. I suggest that the values of parameters used for each figure be clearly stated in the caption (for instance as a tuple of value). Right now, this information is not practical for the reader to obtain.

>>> We now repeat the specifics of synthetic data generation in all main text figures.

14. Figures 5 is very difficult to parse due to the number of curves. An idea to make results more readable (but the authors may think of a better one) would be to (i) remove results on C, which at this point has been shown to be worse than ICOD, and (ii) have three subplot for the three values of μ, each with three curves (ICOD, SCA, conservation).

>>> We considered this suggestion for Figure 5 (now Figure II of the Supplementary Appendix), but we did not manage to find a solution that was really more satisfactory - in particular because we wanted to retain consistency with Figures III, IV and V. Thus we left it as it was before - except that it is now in the Supplementary Appendix.

15. Figure 6: removing the markers might make it easier to distinguish the dashed and solid lines.

>>> We considered this suggestion for Figure 6 (now Figure VII of the Supplementary Appendix), but we wanted to retain consistency with Figures II, III, IV and V. Thus we left it as it was before - except that it is now in the Supplementary Appendix. Besides, we previously had comments from other readers that markers were useful for people with different forms of color vision deficiency, as well as for people who print in black and white.

Minor comments, typos

- I could not understand how parameters Di are chosen, although I may have missed the paragraph that explains it?

  >>> This was mentioned in the caption of Figure 2, but not elsewhere, and we apologize for being slightly cryptic. We now repeat the specifics of synthetic data generation in all main text figures.

- Abstract, “Modeling showed that natural selection on an additive functional trait ...”. Maybe this should be “nonlinear selection”?

  >>> We thank the reviewer for noticing this unfortunate typo. We corrected it.

- first page, “...which have are associated to a functional role...”

  >>> We thank the reviewer for noticing this unfortunate typo. We corrected it.

- page 4, “... the ICOD matrix and its the spectrum without phylogeny ...”

  >>> We thank the reviewer for noticing this unfortunate typo. We corrected it.

- page 13 “... we can also generate sequences wit phylogeny and no selection ...”

  >>> We thank the reviewer for noticing this unfortunate typo. We corrected it.
